# The telomere complex and the origin of the cancer stem cell

**DOI:** 10.1186/s40364-021-00339-z

**Published:** 2021-11-04

**Authors:** A. Torres-Montaner

**Affiliations:** 1 Department of Pathology, Queen’s Hospital, Rom Valley Way, London, Romford RM7 OAG UK; 2 Departamento de Bioquímica y Biología Molecular, Facultad de Ciencias, Universidad de Cádiz, 11510 Puerto Real, Cádiz, Spain

**Keywords:** EndoG, Telomere biology disorders, Hematopoietic stem cells, Telomere-associated secretoy phenotype (TASP), Senescence-associated secretoy phenotype (SASP), Cell of cancer origin

## Abstract

Exquisite regulation of telomere length is essential for the preservation of the lifetime function and self-renewal of stem cells. However, multiple oncogenic pathways converge on induction of telomere attrition or telomerase overexpression and these events can by themselves trigger malignant transformation. Activation of NFκB, the outcome of telomere complex damage, is present in leukemia stem cells but absent in normal stem cells and can activate DOT1L which has been linked to MLL-fusion leukemias. Tumors that arise from cells of early and late developmental stages appear to follow two different oncogenic routes in which the role of telomere and telomerase signaling might be differentially involved. In contrast, direct malignant transformation of stem cells appears to be extremely rare. This suggests an inherent resistance of stem cells to cancer transformation which could be linked to a stem cell’specific mechanism of telomere maintenance. However, tumor protection of normal stem cells could also be conferred by cell extrinsic mechanisms.

## Introduction

There is, presently, substantial evidence that cancers are organized in a hierarchical fashion where a subpopulation of cells called cancer stem cells have the exclusive capacity of initiating and maintaining tumor growth. Similar to normal tissue stem cells, cancer stem cells give rise to self-renewal and non-self-renewal progeny with limited differentiation ability [1, 2]. It has been assumed that the property of self-renewal must be directly conveyed to the cancer stem cell by a normal stem cell without an intermediary non-self-renewing cell. Alternatively, self-renewal ability could arise within a committed cell. In this case a complete or partial stem cell phenotype should be re-expressed concurrently with self-renewal. The present paper reviews a large body of the literature that indicates that, at least, a majority of cancers have an origin in committed rather than normal stem cells and postulates a molecular mechanism underlying this bias in favor of committed cells as cancer targets. This slight change of perspective on the cell of cancer origin accommodates some paradoxical experimental findings in cancer research that suggest an inherent resistance of stem cells to cancer development.

Several endeavors to estimate the replication frequency of HSC (hematopoietic stem cell) have found ample dissimilar figures. One report using 5-bromodeoxyuridine (BrdU) labeling concluded that all HSCs divide every month [3] whereas mathematical modeling led to a very different result: an HSC divides once in every 1–2 years**)** [4, 5].

This difference may be imputed to the assumption of a similar rate of telomere shortening in stem and post-stem cells made in mathematical models or even two assumptions, namely, that the number of divisions between HSC and mature progeny are identical throughout life and that telomere loss is constant in every cell division. These assumptions may be unwarranted, as BrdU labeling shows, mainly because they do not take into account differences in a repair process which is likely to be more efficient in stem cells since expression of telomerase is almost a specific feature of stem cells even if it can be activated occasionally in committed cells. A candidate to provide a differential coupling of telomere repair and cell division in stem versus committed cells is the transcription factor activated protein (AP-1), a heterodimeric complex of the Jun (c-Jun, JunB, or JunD) and Fos (c-Fos, FosB, Fra-1 or Fra-2) family proteins, which can regulate TERT (telomerase reverse transcriptase) expression in human cells [6]. Alternatively, an indirect mechanism of telomere regulation in stem cells could exist even independently of telomerase expression as suggested by the finding that mouse embryonic stem cells can continuously proliferate without undergoing terminal differentiation in the presence of LIF leukemia inhibitory factor (LIF) and its downstream target STAT3 [7]. Still more likely is the involvement of the polycomb group gene Bmi-1. Bmi-1 has been shown to play a key role in maintaining indefinite self-renewal of normal stem and cancer stem cells, a function which must necessarily be linked to maintenance of telomeres in stem cells (discussed below). In summary, it can be safely postulated that HSCs posses a specific or, at least, a more efficient mechanism for maintenance of telomere length than poststem cells. In agreement with this concept germ line and stem cells preserve telomere length along the life of the individual even though some length reduction occurs with advancing age whereas telomere size reduction with age is more pronounced in somatic cells [8]. Furthermore, the rate of telomere reduction has been found to be particularly high in post-stem cells in the first years of life [9]. On the other hand, consistently reduced telomere length independent of age has been observed in cancer cells. Moreover, it was estimated that premature telomere loss preceded leukemia onset for years [10]. This study found telomere shortening at diagnosis in 49 acute myeloid leukemia patients compared to age-adjusted controls. In the study of Engelhardt et al., median telomere length in acute myeloid leukemia was only 1.2kbp shorter than age-matched controls but this shortening took place in spite of an 18-fold increase in telomerase activity [11]. Both telomere size reduction and increased telomerase activity (TA) have been associated to cancer transformation [12]. However, estimation of TA as a feature of cancer is rendered more difficult by the association of TA with normal cell proliferation in addition to cancer. For instance in the hematopoietic tissue telomerase is suppressed in quiescent CD34+/CD38- (quiescent) stem cells and is upregulated upon entering the cell cycle and with expansion of the CD34+/CD38+ compartment [8]. The apparent contradiction pertaining to cancer cells (as compared to normal stem cells) of telomere erosion in the presence of increased TA can be solved by postulating independent regulation of telomere length and telomerase expression (or other cooperating factors in telomere maintenance) in normal stem versus somatic and cancer cells. In other words, a feedback regulatory loop connecting telomere erosion and telomerase activity (or other putative telomere maintenance machinery) would be more efficient in stem cells than somatic or cancer stem cells. Telomere length reduction and increased TA activity occur in many common adult cancers and leukemias [1] but the extent of their involvement is so disparate that it suggests an abnormal lack of coordination in order to accomplish telomere repair. For instance, in chronic myelogenous leukemia (CML) no statistically significant difference in TA activity between CML and control cells was detected after correction for the fraction of cells in G0 phase [8]. Telomere length was 1 kb shorter than age-adjusted controls [8] in Ph + cells as compared to Ph- non leukemic cells but the rate of telomere loss has been reported to increase with disease progression from chronic phase (CP) to acute phase (AP) [13]. In keeping with a perturbed regulatory telomere-telomerase loop, TA was significantly lower in CML CD34+ cells than in normal CD34+ cells. Nevertheless, up to a 50% increase in TA has been observed in more than 50% of patients in blast crisis [8]. TA activity is especially prominent in some tumors such as acute leukemias, BP (blastic phase) of CML and small cell lung tumors [12].

The idea that short telomeres of cancer reflect the age of the tumor cannot be maintained since this would lead to auto-extinction of the neoplastic clone. Therefore the shorter telomeres of cancer cells as compared to age-adjusted controls suggest a cell of cancer origin in the post-stem cell compartments consistent with telomere length stabilization seen after crisis [14] (and likely after transformation). On the other hand, acquisition of self-renewal in cancer development seems to be concurrent with re-expression or overexpression of telomerase. These considerations suggest that final cancer transformation of acute leukemia occurs in a post-stem cell that had undergone some degree of telomere erosion and subsequent reactivation of telomerase, or alternative lengthening of telomeres,(ALT).

### Widespread occurrence of telomere erosion preceding cancer transformation

In a former paper [15], I pointed out the parallelism between telomerase re-expression preceding cancer development in vivo with the in vitro emergence of cancer stem cells after crisis. In that paper, two tumor categories were delineated according to the presumed stage of cancer cell origin: childhood tumors/acute leukemias arising from immature cells and the common adult cancers presumably arising from transformation of more differentiated cells. In this type of cancer, a prolonged pre-neoplastic phase allows for accumulation of somatic mutations and severe telomere erosion with possible activation of p53 (or lack of it through loss of function) [15]. TP53 mutation may appear stochastically without oncogenic transformation as mutation in any other gene. However, loss of p53 function being already in place may be critical in cooperation with subsequent mutations to trigger transformation. The process must be quite different in acute leukemias and other acute cancers (frequently occurring in infancy) which contain fewer mutations and less common involvement of p53. Epidemiologic data lends further significance to the division in these two tumor groups since, as expected, common cancers affect mainly adult patients whereas acute cancers presumably caused by a few driver mutations can affect both children and adults.

A survey of the literature sheds strong support for the involvement of a mechanism of telomere attrition in common adult cancer.

For instance, in pancreas [16] telomere length reduction is the most prevalent and early preneoplastic-associated finding. In breast, a very precise correlation of the telomere erosion pathway of oncogenesis with histopathologic changes exhibited by mammary epithelium in its transition from usual ductal hyperplasia (UDH) to ductal carcinoma in situ (DCIS) to invasive carcinoma was described by Chin and coworkers [17]. Progressive changes in telomere length reduction, increased genomic instability and re-expression of telomerase correlated with the histological features which characterize these entities (UDH, DCIS and invasive carcinoma). Other reports confirm that telomere shortening is prevalent in breast lesions of ductal carcinoma in situ [18].

Squamous carcinoma of esophagus has been reported to arise from a telomere-shortened epithelial field [19]**.** In the intestine, increased telomerase expression correlated with development of colorectal carcinogenesis from normal mucosa to small, intermediate and adenomatous polyps and invasive carcinoma [20]. Telomerase activity increases during progression from melanocytic naevi to malignant melanomas [21].

Telomere length reduction could also be present in case of tissue atrophy at the level of the stem cell and could underlie the observed association of cancer with several atrophic conditions (or conditions associated with HSC exhaustion discussed below) [22]. However, in spite of the usual finding of telomere erosion in tumors of early cells such as acute leukemias/lymphomas, the involvement of such mechanism in their pathogenesis seemed less tenable as a substantial fraction of these malignancies are characterized by early onset, immature cell morphology, diploid karyotype and absence of structural chromosomal aberrations (except those specific translocations that happen to affect genes regulating hematopoietic development). Cancer transformation of cells from early differentiation stages were less likely to have any connection with telomere damage as the prevalent consensus was that telomere erosion is associated to cancer only indirectly by means of the genetic instability provoked as a result of chromosome uncapping [23].

### Telomere damage signaling may follow different pathways in tumors of immature versus mature cells

In the following pages a large body of literature will be presented that strongly suggests the involvement of the telomere complex machinery in tumors of early cells such as acute leukemias. These tumors, however, may use not exactly the same telomere signaling circuits, differing perhaps in sequence of signals or telomerase overexpression instead of telomeric signaling which might be preferentially used by common adult cancers. The complex interplay between the p53, p16 pathways and telomerase expression (and other genes) in overcoming senescence is beyond the scope of this essay. However, the generation of some knock-in p53 mutants has allowed the dissection of p53 suppressor functions that support the concept of different oncogenic pathways followed by tumors presumably arising from mature versus immature stages of development. The p53R172P mutation carrying the same aminoacid substitution found in some human tumors is defective in inducing apoptosis but weakly induces p21 and cell cycle arrest in response to γ-radiation. It was shown that mice homozygous for this mutation are protected from early onset T cell lymphomas characteristic of p53-null mice suggesting that apoptosis does not play any role in protection against early onset thymic lymphomas. Moreover, tumors that, eventually, arose in these mice displayed the phenotype of early progenitors. They have a diploid DNA content and lack uneuploidy observed in Trp53-null mice. By histological analysis many of them were identified as histiocytic lymphomas or high-grade lymphomas with frequent double staining for CD4 and B-cell markers as well as some high-grade sarcomas including osteosarcoma and rhabdomyosarcoma. This is in contrast to tumors that develop in Trp53-null mice which are predominantly thymic lymphomas that stain positive for CD4 and CD8. With all the caveats implied in the extrapolation of telomere biology of mice to humans due to the long telomeres of mice, these experiments demonstrated that apoptosis, which is believed to be associated to tumors arising after severe telomere attrition, is dispensable for suppression of spontaneous tumors of early cells [24].

The different oncogenic routes followed by transformation of early-immature cells versus cells of more advanced stages of differentiation may explain why p53 loss of function is associated also, although not as frequently as in common adult cancers to acute leukemias that, interestingly, contain a different spectrum of p53 mutations (for instance, nonmutational wtp53 inactivation predominates in AML (acute myeloid leukemia) [25]. Accordingly, p53 functions in normal hematopoiesis are also dependent on stage of development. For instance, in the hematopoietic stem cell compartment, p53 regulates stem cell quiescence, apparently through its targets Necdin and Gfi-1 but independently of p21, and its deficiency promotes HSC mobilization, reducing the stem cell pool and increasing post-stem cells which may lead to HSC exhaustion [26, 27]. The higher frequency of tumors of late cell origin in the epithelial tissue (in contrast to mice where the incidence of lymphomas and sarcomas exceeds greatly that of epithelial cancers) is in line with the role of long telomere length protecting from severe telomere attrition and aneuploidy linked to this oncogenic pathway but could also be due to differences in epithelial biology. For instance, Dickson et al [28] reported that hTERT transduced human keratinocytes entered a slow growth phase after reaching the normal population doublings from which rapidly dividing cells emerged that were deficient in p16 expression. However, normal growth control mechanism and differentiation remained unaffected. Likewise, keratinocytes transduced with cdk4R (a point mutant of cdk4 unable to bind p16 which neverteless retains cyclin-dependent kinase activity) and p53 dominant negative (DN) mutation that had been immortalized by hTERT stratified and underwent an epidermal suprabasal differentiation [29]. These experiments suggest that differentiation may proceed unperturbed in epithelial tissues allowing for severe telomere loss before immortalization.

In any case, escape from senescence and associated constitutive telomerase re-expression afforded by p53 deficiency probably represents a final event in transformation in tumors of mature and immature cell origin and could be conferred by canonical and non-canonical p53 mutations alike.

Evidently, the association of well differentiated epithelial and late onset leukemias from more advanced stages of differentiation with telomere damage and genetic instability and that of early cell leukemias with normal karyotypes is an oversimplification. Numerous cytogenetic alterations, including complex karyotypic alterations, are found in leukemias but most of them are recurrent, non-random alterations that affect specific genes that deregulate distinct steps in the complex process of hematopoietic development. Taking this fact into account as well as the possibility of genetic instability unrelated to the telomere, the above association appears to have biological significance and the same could apply to other pediatric and undifferentiated tumors [15]. In fact, 45 % of all AML patients have no cytogenetic alterations [30].

Telomere erosion signaling is a double-edge sword. Telomere-induced senescence is considered to be a mechanism to counteract genetic instability and tumor growth. Nevertheless, after massive apoptosis induced by severe telomere shortening in crisis some cells emerge with the phenotype of immortal cancer cells. There is an analogy between the in vitro emergence of immortal cells after crisis and in vivo development of cancer but the inference of any similitude between these pathways seems to be disallowed by the extremely low efficiency of transformation in vitro after crisis. However, this view may change thanks to new experiments that have unveiled the opposite roles played by senescence under in vitro and in vivo conditions. These experiments have shown that the tumor promoting effect of senescence in vivo could make up for the low efficiency suggested by in vitro transformation after crisis. L. Mosteiro et al. [31] observed a discrepancy between in vivo and in vitro reprogramming (using teratoma formation as an assay for pluripotency) in mice carrying a doxycycline inducible OSKM transgene (reprogramming factors: OCT4, SOX2, KLF4 and c-Myc) (i4F, for inducible four factors) combined with heterozygous null alleles for p53 or Ink4a/Arf. Heterozygous mice were used due to the short age of the homozygous mice. The efficiency of reprogramming as measured by teratoma formation was i4F;p53 null > i4F > i4F;Ink4a/Arf null. The higher reprogramming efficiency of p53-null and i4F strains than Ink4a/Arf null was in striking contrast with in vitro findings where Ink4a/Arf null shows maximal reprogramming efficiency. This was shown to be due to differences in the senescent response associated to each of these strains which is weaker in the Ink4a/Arf null in vivo. They described a senescent associated secretory phenotype (SASP) that promotes in vivo reprogramming characterized by upregulation of cytokines and inflammatory factors including Il-6, tumor necrosis factor-α (TNFα) and the nuclear factor-κB (NF-κB). The senescence phenotype as well as OSKM-induced reprogramming was enhanced in cells deficient for the telomerase RNA compoment (Terc-null mice) (G2 Terc-null) due to absence of telomerase activity leading to short telomeres and a premature aging phenotype. Previous to these experiments Braig et al. had shown that a moderate level of telomere erosion can confer a selective growth advantage [32]. Using a murine BCR-ABL^+^ cell culture model from bone marrow cells of telomerase knockout mice (mTR^−/−^) with pre-shortened (generation G2) and critically short (generation G4) telomeres as well as from wild type animals with normal telomeres (mTR^+/+^), they disclosed a remarkable difference in proliferation kinetics between cells with moderately shortened telomeres (generation G2) and cells with critically short telomeres (generation G4). As expected, cellular senescence was observed in the latter (G4) cell cultures. However, cells with moderately short telomeres underwent a growth-supporting inflammatory response involving up-regulation of multiple pro-proliferative mRNAs which they described as a telomere-associated-secretory phenotype (TASP). Whereas late generation (mTR−/−) mice were shown to display aneuploidy and end to end chromosomal fusions, no chromosomal gains or losses was observed in cells from mice with moderately shortened telomeres (G2). The expression of cytokines and other components of TASP increased during transition from the chronic phase of CML (CP) to acute phase of CML (AP). The transcriptomes of the senescence-associated-secretory-phenotype (SASP) described by Mosteiro et al. and that of TASP overlap partially. In the former, the role of IL-6 through the JAK/STAT pathway and NFκB activation through p65 phosporylation was shown to be paramount. Reinforcement of NFκB activation was also detected in TASP whereas the senescent response of G4 cell cultures in Braig experiments showed downregulation of proliferation-promoting genes and upregulation of inflammatory genes including IL-6 and IL1α.

**Fig. 1:**
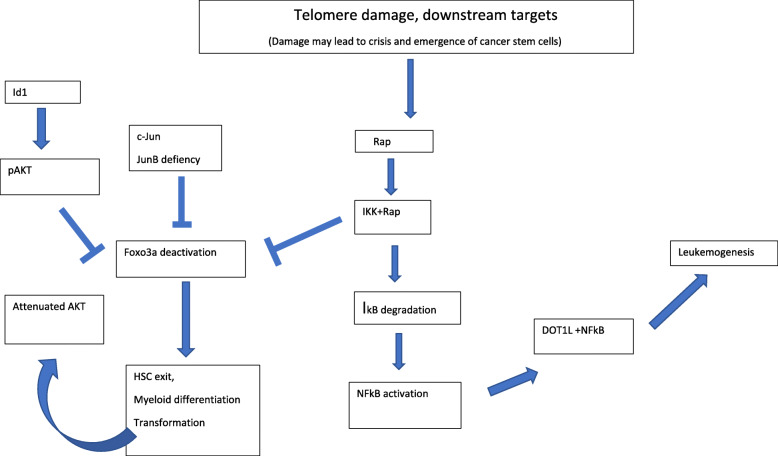
Telomere damage, downstream targets. Damage may lead to crisis and emergence of cancer stem cells

**Fig. 2:**
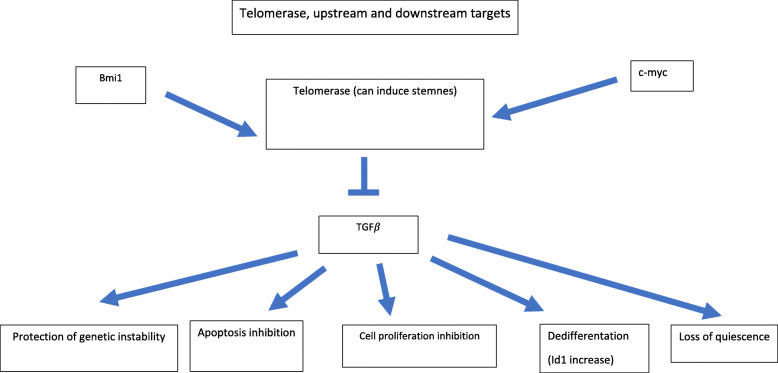
Telomerase, upstream and downstream targets

The two kinds of telomere-associated response bear an interesting parallelism with our two tumor categories. A pro-tumorigenic signal delivered by telomere damage could be involved in both tumor types but threshold of telomere erosion required and, possibly, other concurrent factors may be different for each of them. **(**Figs. 1 and 2**: telomere and telomerase pathways.**

An element of TASP (as well as of SASP) is the upregulation of NF-κB by RAP1, a component of the shelterin complex of telomere binding proteins. This finding is particularly significant because it is one of the specific features of the leukemia stem cell phenotype, absent in HSCs [1, 2]. Rap1 associates with telomeres indirectly through interaction with the sheltering complex member TRF2 but its telomeric functions are not well known. However, Rap1 is also present in the cytoplasm, unassociated to TRF2 and can participate in non-telomeric functions such as mitochondrial metabolism and inflammation [33]. Therefore, Rap1 is a good candidate for coupling of telomere alterations to oncogenic signaling pathways. In fact, it has been shown that Rap1 binds to the IKK complex promoting degradation of the NFκB inhibitor IκB, which results in nuclear translocation and activation of NFκB [34]. In addition, IKK may promote nuclear exclusion and inactivation of FOXO3a although the involvement of Rap1 in FOXO3a inactivation has not, to our knowledge, been studied. This function is also independently accomplished by p-AKT and has been found repeatedly associated with enhanced cell proliferation and tumorigenesis. In a revision of 113 human primary breast tumor specimens, a correlation was found by IHC (immunohistochemical staining) between positive IKK and p-AKT and cytoplasmic FOXO3a in 90cases [35].

### The leukemia stem cell phenotype

The origin of cancer directly from the normal stem cell was mainly supported by reports that identified the CD34+/CD38- tumor subpopulation as the fraction that can initiate leukemia upon transfer to nonobese diabetic-severe combined immunodeficiency (NOD/SCID) recipient mice. Normal human HSCs endowed with full repopulating ability are also found within the CD34+/CD38- fraction of human hematopoietic cells. However, later findings indicate that Fc-mediated clearance of anti-CD38 conjugated cells was responsible for the low leukemia initiating cell (LIC) activity that had been observed in the CD34+/CD38+ fraction. Taking this fact into account, LIC activity would lie, in most AML cases within the CD34+/CD38+ progenitor population [36]. CD34+/CD38+ cells are apparently derived from the CD34+/CD38- subpopulation. Some markers such as HLA-DR, CD71, Sca-1+ and Lin- are shared by HSCs and leukemia stem cells (LSCs). However, there are also significant differences in the phenotype of LSCs and HSCs [1, 2]. Normal HSCs exhibit lower levels of CD47 than LSCs. CD33 is variably expressed by LSCs but its expression is lacking in HSCs. The adhesion receptor CD96 has been described in LSCs from T-acute lymphoblastic lymphoma (T-ALL) and AML but only in a minority of normal HSCs. IREM-1 and C-type lectin-like molecule has been found differentially expressed on AML stem cells and HSCs. Thy-1(CD90) is downregulated as normal HSCs progress to downstream progeny whereas LSCs exhibit low Thy-1 expression. Conversely, c-Kit is more strongly expressed in LSCs than in normal HSCs. A unique marker of LSCs not expressed by HSCs is the IL-3 receptor (CD123) which was found to be expressed on 98% of the CD34 + CD38- cells of 16 AML patients but was undetectable in normal CD34 + CD38- cells. NFκB is constitutively activated in the majority of primary AML samples including the quiescent cell fraction but is not activated in normal HSCs [1, 2]. The expression of this molecule in LSCs may be especially significant since that expression would be one of the consequences of TASP-mediated transformation.

### Tumor promoting effects of the telomere complex machinery independent of telomere length attrition

The expression of the growth-promoting telomere-associated-secretory-phenotype (TASP) and its hypothetical role on tumor development appears to be the consequence of a moderate level of telomere erosion induced in G2 cells (generation2, telomerase null) which is independent of telomerase activity as the results obtained in TERT knockout mice show. However, some of the observations described in this and preceding papers imply that tumor promoting effects triggered by telomere damage in vivo may also involve the interconnected TERT and Terc compensatory response. In fact, there is overwhelming evidence in support of a role of hTERT on cancer promotion independent of telomere protection [37].

Furthermore, it has been reported that normal and neoplastic non-stem cancer cells can be converted to a stem-like state by transfection of hTERT [38].

### The telomerase oncogenic pathway

Some insights into the oncogenic pathway triggered by telomerase have been contributed by several researchers. One important target of telomerase is the transforming growth factor receptor β (TGFβ). Telomerase abrogates the growth inhibitory effect of TGFβ. In mouse embryonic fibroblasts (MEFs) it was found that 86% (18/21) growth-inhibitory genes suppressed by mTERT were related to the TGFβ signaling pathway [39].

TGFβ suppression may have oncogenic effects even in the absence of effects on cell proliferation. This was shown in the breast cell line Cah1 by introduction of a dominant negative TGFβ type II receptor (Ca1h DNR). Effects on cell proliferation in Ca1h DNR were mild and some cell cycle genes changed even in an opposite direction than expected (c-myc was lower than in Ca1h cells and p27 was increased). However, TGFβ suppression had a dedifferentiating effect as shown by a predominance of basal cells compared to luminal cells and corresponding increase in cytokeratin markers of basal type. Reversion to a more immature phenotype was associated with Id1 overexpression. However, forced expression of Id1 did not enhance tumorsphere formation, size of side population (SP) fraction or tumor formation in vivo, suggesting that Id1 acts upon the progenitor proliferating population rather than on stem or cancer stem cells [40]. Increased telomerase would be expected to occur concomitantly to TGFβ suppression and affect the same cellular compartment.

Telomerase displays other functions relevant for tumorigenesis like protection from apoptosis [41, 42], (it may antagonize apoptosis induced by p53), activation of quiescent stem cells and induction of HSC mobilization, a property shared with p53 loss of function mutations and c-myc [26, 43] as well as protection of genetic stability which may help to explain the diploid karyotype of many acute leukemias [42]**.** Some of the functions of the c-myc oncogene (and other oncogenes like Bmi-1) could activate the telomerase signaling pathway either through the ability of c-myc to induce telomerase expression [44] or indirectly, through the ability to increase cell cycling and consequently telomere erosion although the latter could be, at least partially, cancelled by the former. In addition, constitutive expression of c-myc can be more oncogenic when expressed in a chronologically inappropriate developmental stage as demonstrated by c-myc expression under the control of Ig enhancers in Burkitt lymphoma or the t(14;15) chromosomal translocation involving the T cell receptor α (Tcra) and c-myc loci in T-ALL induced by Pten deficiency which is similar to a t(8;14) associated with a subset of human T-ALL [45] to be discussed later).

### Telomere erosion must be accelerated when cells leave the stem cell compartment. A favorable ground for neoplastic development

A stem cell mechanism present in stem cells for protection of telomere length (and/or specific silencing of the oncogenic ability of telomerase), which seems to be required to explain the lifetime function of stem cells should be either relaxed or lost in post-stem cells. Therefore, cells engaged in tissue turnover not constrained by the need to preserve indefinite self-renewal are bound to undergo progressive telomere attrition and become vulnerable to telomere attrition-induced oncogenic pathways. Such pathways should significantly spare stem cells reducing the incidence of stem cell tumors. I hypothesize that loss or impairment of this protective mechanism is concomitant with the loss of indefinite self-renewal. This oncogenic pathway is compatible with the concept that an initial event may occur in a stem cell which, nevertheless, will not be transformed until it has engaged in tissue turnover and undergone sufficient telomere erosion or an alternative trigger of telomerase re-expression. Even if telomere erosion would start in stem cells as in tissue atrophy or HSC exhaustion, a critical level of telomere erosion would occur probably only in downstream stages (at least in most cases). A high prevalence of this pathway of tumorigenesis predicts a low occurrence of true stem cell tumors. Evidently, a low incidence of stem cell tumors might also be due to a stem cell-specific resistance to tumorigenesis (or both).

### Leukemia and hematopoietic development. Descriptive studies

The following overview of the literature is intended to provide a framework to contextualize leukemic transformation in the course of hematopoietic development. Several common themes run through most of the observations discussed:

1/ A pure stem cell neoplastic phenotype is extremely rare, i.e. practically all leukemias belong into a lineage restricted compartment. The phenotypes of leukemia stem cells and HSCs overlap only partially. Considering the early loss of self-renewal capacity in normal hematopoietic development (apparently by, or, anterior to the multipotential precursor stage, MPP) most if not all leukemias can be ascribed into a lineage restricted compartment. 2/ tumors arise most often in relation to developmental blocks and/or conditions that can favor replication stress and telomere shortening. Developmental blocks may occur at any step of the differentiation ladder but in general are located well downstream of the loss of self-renewal capacity and bestow a tumor phenotype akin to that of the corresponding normal stage of development 3/ Pre-existing telomere shortening has been reported in non-clonal hematopoietic stem cells of patients with de novo AML [10] as well as in patients with early T-cell leukemias (see below). Telomere erosion is a natural consequence of cell proliferation but there are some situations in hematopoietic development characterized by unscheduled proliferation where replication stress and, conceivably, telomere erosion could be accelerated. These conditions are frequently associated to leukemia development. 4/ finally, leukemia is often associated to HSC exhaustion. The main causes of HSC exhaustion are directly related to mutations in telomerase components (i.e. dyskeratosis congenita (due to mutation of the RNA component of telomerase (TERC) or a hereditable heterozygous mutation in TERT responsible for autosomal dominant aplastic anemia) [46] and may lead to accelerated telomere erosion. Accelerated telomere erosion is also a feature shared by syndromes of premature aging and Down syndrome, all of them associated to increased cancer incidence [46].

### Extremely low incidence of true undifferentiated leukemias

In clinical classifications of leukemias the category of true undifferentiated leukemias is undefined. Two papers dealing with classification of hematologic malignancies based on flow cytometry show the extreme rarity of leukemias with a stem cell phenotype. The first [47] estimated that acute undifferentiated leukemias comprise about 1% of acute leukemias and that this figure could still be reduced by the use of some cytoplasmic and surface markers. In a later paper [48] the group of undifferentiated acute leukemias was dropped. (incidentally it is well known that stem cells present at the level of the bone marrow niche possess not only potentiality for hematopoietic differentiation but also osteoclast differentiation potential. It is noteworthy that this type of differentiation is never manifested by leukemias which would be expected in the assumption of a direct transformation of the HSC [see below).

### Early loss of indefinite self-renewal ability

HSC can be divided into long-term HSCs (LT-HSC) which have permanent self-renewal ability and short-term HSCs (STHSC) with more limited self-renewal. ST-HSC give rise to non-self-renewing multipotential progenitors (MPP) [7, 49]. The first decisions into lineage diversification are taken within the MPPs through the process of lineage priming. This is associated with upregulation of a set of transcription factors already present in hematopoietic stem cells that enforce the adoption of one of the lineage branches while repressing the alternative option. The first branching point decision appears to be concomitant or even posterior to the loss of indefinite self-renewal which appears to be lost already in MPPs or even earlier as ST-HSCs have diminished repopulation potential compared to LT-HSC [49]. Transcription factors (TFs) expressed in HSCs and MPPs important for the first steps of lineage diversification include IKZF1 (Ikaros), E2A (Tcf3), PU.1(Spi1), GATA-1, C/EBPα and others [50]. These TFs are also involved in later fate decisions. The phenotypes corresponding to these later stages are well represented within clinical and model tumors but do not seem to be associated to the stem cell phenotype. Why deregulated expression of transcription factors that are expressed in HSCs is associated with leukemias/lymphomas that display neoplastic phenotypes corresponding to committed cells but not the HSC stage phenotype?. One reason for the extreme rarity of the pure stem cell phenotype could be that a complete block at the initial step of development is unlikely to take place as exit cells have several routes of development available and can take any route if another is prevented. However, as will be discussed below, partial blocks could be more efficient than complete blocks in promoting leukemogenesis. In addition, this answer raises another question: Does tumor development require exit from the stem cell pool and, then, why developmental arrest predisposes to tumor formation? Is engagement in tissue turnover and telomere erosion derived from it, a condition for immortalization? Can developmental blocks accelerate telomere damage?

### Correlation of hematopoietic developmental blocks and leukemogenesis

HSCs comprised two populations according to their repopulation potential: long term HSCs and short term HSCs. Interestingly, LT-HSCs have been demonstrated to lack expression of CD34 as well as the cytokine tyrosine kinase receptor Flt3 whereas ST-HSC are LSKCD34 + Flt3- [51].. Downstream of HSC and MPP, the lymphomyeloid progenitor (LMPP) is considered the first major restriction point of diversification between lymphomyeloid and erythroid pathways [51]. Nevertheless, the older canonical hierarchical scheme of hematopoietic development postulates also a myelo-erythroid stage that cannot be discarded [51]. Paradoxically, according to Dias et al. Ikaros is dispensable for the generation of LMPPs but required for high expression of Flt3 and subsequent lymphocyte differentiation [52]. HSCs in Ikaros-null mice lack the Flt3 tyrosine kinase normally upregulated during transition from ST-HSC to LMPP. Ikaros null mice lack all B, NK (natural killer cell) and fetal T cells although a small number of early T cell progenitors can be detected in the thymus of these mice. Myeloid differentiation is not impaired [53]. The LSK phenotype (lineage -, Sca-1 +, c-Kit +) is detected in HSC and MPPs. Expression of Flt3 in LSK has been instrumental in the characterization of the LMPP branch with lymphomyeloid but not megakaryocytic and erythroid potential [51]. However, lack of Ikaros at a later stage, in the common myeloid progenitor (CMP) results in increase megakaryocyte-erythrocyte progenitors (MEPS) at the expense of myeloid progenitors [53]. Ikaros has been identified as a target of the E2A protein, E47. In E47 deficient mice, HSCs compartment appears normal but downstream population MPP is reduced in number and lymphoid differentiation is compromised [54]. E2A proteins are involved in development of LMPP and restriction of megakaryoblast-erythoblast and myeloid differentiation in LMPPs [52]. The role of E2A proteins in promoting lymphoid and repressing myeloid lineage differentiation is also manifested by the opposite role played by its competitor Id1 (a member of the helix-loop-helix family of TFs that antagonize E2A proteins by inhibiting their ability to bind DNA). Id1 is highly expressed in common myeloid progenitors. When bone marrow (BM) cells were transduced with an Id1-expressing MSCV retrovirus cells could divide for over 1 year in culture in the presence of stem cell factor. The majority of cells were myeloblasts with some promyelocytes and myelocytes. These cells could be induced to differentiate with physiological inducers of myeloid differentiation. Transplantation of cultured cells to mice induced a myeloproliferative (MPD)-like disease. However, secondary recipients did not develop leukemia [50].

The expression of Ikaros at the earliest stages of hematopoietic development where it induces expression of lymphoid genes while repressing stem cell specific genes [55] suggests that the absence of Ikaros might divert hematopoietic differentiation away from lymphoid lineage and, concomitantly, preserve the indefinite self-renewal of HSCs. This feature makes Ikaros inactivating mutation an ideal candidate for direct malignant transformation of HSCs and therefore this mutation should be expected to be prevalent among stem cell leukemias. Nevertheless, Ikaros is also involved at later stages of development and accumulated data indicate that Ikaros mutations are always associated to lineage restricted leukemias. A germ-line homozygous mutation in the Ikaros DNA-binding domain of mice has been described associated with absence of T, B and NK lineages. Most mice died within 4 weeks from infections or cannibalism but stem cell tumors were not reported. Thus, a complete block at this stage does not seem to lead to tumor formation [56]. In LMPPs, Ikaros limits transcripts to the lymphoid and myeloid programs whereas lack of functional Ikaros in common lymphoid progenitors (CLPs) leads to preferential development of natural killer (NK) cells at the expense of T and B lymphocytes [57]. IKZ1 heterozygous mice develop a partial arrest in a rather advanced stage of T cell differentiation as attested by the accumulation of T cells with abnormal single and double CD4, CD8 profile and altered TCR (T-cell receptor) expression. The arrested cells show greatly enhanced proliferation (200 fold increased (3H) thymidine incorporation compared to 7.7 of wild type (wt) cells after TCR stimulation in vitro) which evidently should favor telomere erosion. After 3 months 100% of mice developed a T cell lymphoma/leukemia with rearranged TCRβ and either CD4 or CD8 surface expression. Loss of heterozygosity (LOH) for the Ikaros mutation was detected in leukemic cells [58]. Some leakage of the block must be required for leukemic transformation as the appearance of the T lymphoid phenotype shows. In human patients, IKZF1 (the Ikaros gene) is deleted frequently in B cell precursor leukemia [59] and has also been found associated to T, B and myeloid combined immunodeficiency [59]. IKZF is deleted in 15% of paediatric B-cell precursor acute lymphoblastic leukemia. It is not uncommonly seen in chronic myeloid leukemia (CML) in lymphoid blast crisis [60]. In mice, reduced Ikaros expression results in a differentiation block at an early pro-B cell stage [60].

The E2A gene encodes two spliced variants: E12 and E47 but E47 is the major player and recapitulates the phenotype of E2A. Yang et al. [54] found grossly normal numbers of HSCs defined as CD150+ CD48- but a 50–70% reduction of non-renewing MPPs (CD150- CD48-) and downstream populations in E47KO (E47 knockout) mice. MPPs failed to upregulate Flk2/Flt3 or initiate V(D) J recombination. Early thymic progenitors (ETPs) were reduced 4 fold in E47+/− In addition E2A deficiency is associated with depletion of erythroid progenitors and impaired HSC self-renewal ability mediated by decreased levels of p21 and Gfi-1 as well as 2-fold decrease in LT-HSC and 4-fold-decrease in MPPs of E2A^−/−^ mice [61]. However, Dias et al. reported only a mild decrease in numbers of HSCs and MPPs but significantly reduced numbers of LMPPs in E2A+/− and E2A−/− mice. They speculated that the decreased frequency of LMPPs does not seem to be due to a requirement of E2A for induction of Flt3 because they did not detect a compensatory increase in Flt3^−^ or Flt3^low^ cells [52]. However, in the absence of TCF3 (E2A), IKZF1, MYB or SPI1 the expression of Flt3 and consequently LMPPs is reduced [62].

An increased inactivation mutation rate of E2A has not been associated, to my knowledge to undifferentiated, stem cell leukemias. In contrast, E2A deficieny was shown to lead to rapid development of T cell lymphomas expressing late markers CD4 and CD8 [63]. E2A together with HEB are needed to maintain the DN3 arrest which is crucial for TCR recombination and completion of the pre-TCR and β checkpoint control. Loss of E2A function at this stage induces unscheduled proliferation resulting in a potent promotion of leukemogenesis [64].

#### E2A and the B lymphocyte differentiation program

The common lymphoid cell stage (CLP) is reached after loss of myeloid potential. It is characterized by Sca1^int^ c-Kit^int^ IL7R^high^ Flt3+. This compartment can be divided into an ALP subset (all lymphoid lineages, including NK, and dendritic (DC) and a BLP subset (B lymphoid precursor), according to the expression of Rag and LY6D [62] Transition to the BLP stage is critically dependent on E2A (TCF3). Although E2A proteins are sufficient to induce characteristic B cell molecules such as IgH and Igκ [65], the initiation of the B cell development program requires the collaboration of Foxo1 as well as STAT5 activation induced by IL7R signaling. These molecules acting in concert elicit the expression of EBF1 [62]. In turn, EBF1 induces Pax5 expression. Pax5 has a minor impact on the earliest progenitors but it forms an autoregulatory loop with EBF1 that maintains B cell lineage fidelity [66]. This fact appears to be responsible for the association of Pax5 deletion with biphenotypic leukemias rather than an earlier cell of origin of the leukemia stem cell [67].

The activity of these genes converge on the formation of the pre-B cell receptor (pre-BCR), composed of the rearranged IgH chain and the surrogate light chains IgLL1 (λ5 in humans) and VPREB plus CD79A and CD79B. These genes are direct targets of EBF1 and/or Pax5. Combined signaling through the pre-BCR and IL7-STAT5 leads to expansion of pro-B and early pre-B cell populations. Then, IL7-STAT5 signaling abates leading to cell proliferation arrest allowing Ig light chain rearrangement [62]. Cells containing cytoplasmic Ig (cIg) are pre-B cells but once an Ig light chain is formed, B cells can express surface Ig and become immature B cells. Transition from pre-B cells to immature B cells may be deranged either through an IL7-STAT5 proliferation stimulus persisting beyond its normal limits or by inability to accomplish Ig recombination. This is usually the result of uncontrolled proliferation or deficiencies in pre-BCR components.. Although the transition from pre-pro B cells to immature B cells reflects the sequential expression of E2A-EBF1-PAX5 as well as pre-BCR signaling, the process is more complex than suggested by this linear pattern due to the involvement of other players including Ikaros family members, the interferon regulatory factors Irf-4 and Irf-8 [68] and Runx1 or Ras [69] and functional crosstalk between these molecules in developmental progression and in activation of components of the pre-BCR complex. Furthermore, changes in expression of these genes can affect B cell development in a dose dependent way, for instance, a complete loss of Ebf1 results in arrest at the pro-B cell stage whereas the pre-B cell population is decreased after the single loss of one allele due to a reduced response to IL7 associated with reduced expression of the pre-BCR component λ5 [66].

Although mutations of the genes participating in pre-BCR signaling are frequently found in leukemias, experimental tumor models show that the greatest oncogenic impact is caused by the combination of signals that induce uncontrolled proliferation with others that disrupt Ig rearrangement and induce a partial block in differentiation. Thus, mutations in E2a, Ebf1, Pax5, Runx1, Ras, Irf4, Irf-8 or Ikaros can be found in a large proportion of pediatric and progenitor leukemias [62]; however, model tumors show that leukemogenesis is more efficient by expressing constitutively active STAT5 (STAT5-CA) in combination with the loss of one allele of Ebf1 or Pax5 which induce a partial block in B cell differentiation. For instance, Stat5b-CA x Ebf1^+/−^ and Stat5b x Pax5^+/−^ transgenic mice develop rapid onset leukemia with complete penetrance whereas leukemogenesis is somewhat less efficient when crossing Stat5b-CA with Rag2^−/−^ or μMT^−/−^ mice. Loss of function of Rag2 or μMT results in a complete differentiation block due to lack of recombination or absence of the immunoglobulin heavy chain, respectively. The phenotype of these mice differs from STAT5b x Ebf1 or Pax5 heterozygotes in a delayed onset and lower penetrance. Microarray analysis showed that a subset of Ebf1 and Pax5 target genes were derepressed including Bcl-2, cmyc (> 11-fold increase in expression) and Tnfsf1, which encodes RANKL, a factor that stimulates B progenitor cell proliferation (> 44-fold increased expression) [67]. Such potent induction of cell proliferation coupled to a delay in the transition through the next developmental stage might, conceivably, deregulate the telomere transcription machinery leading to either telomerase overexpression or telomere shortening (or both).

### A very early developmental block not associated to transformation

Transition to the BLP subset of CLP compartment requires, in addition to E2A other bHLH (basic helix loop helix) proteins as demonstrated by Ikawa et al. These authors managed to arrest cells in an early multipotent stage by overexpression of Id3, a member of the Id family of proteins that antagonize E2A and other bHLH proteins and cultivation under B cell induction conditions [70]. These cells called induced leukocyte stem cells (iLSC) could be maintained almost indefinitely in culture while maintaining capacity for multilineage reconstitution when transferred to irradiated recipients [70]. Malignant transformation was not reported.

### The case of E2a-Pbx1 fusion protein

E2a-Pbx1 protein is formed subsequent to chromosomal translocation 1;19 and has been found frequently associated to pre-B cell leukemia. Unexpectedly, in experimental models designed to study the mechanism by which E2a-Pbx1 induces leukemia, retroviral mediated expression of E2a-Pbx1 in hematopoietic progenitors gives rise to myeloid, not lymphoid leukemia. The work of Woodcroft [71] et al., has clarified this puzzle by showing that the neoplastic phenotype depends on the developmental stage of the cell into which the oncoprotein is introduced. E2a-Pbx1 appears to function as a dominant negative mutation of E2A which, when introduced in early progenitors induces a complete block prior to the CLP stage. In contrast, myeloid and erythroid lineage development are only relatively impaired with deficient maturation from CD11b^+^/Gr^−^ to CD11b^+^/Gr^+^ and a reduction in macrophages and Ter-119^+^. These experiments reinforce the concept that transformation is usually linked to partial, rather than complete, differentiation blocks. Interestingly Hoxa9 which is abundantly expressed in HSCs and subsequently downregulated was identified as the main mediator of the leukemogenic effects of E2a-Pbx1. Obviously, HSCs should be, at least, relatively unharmed by overexpression of this molecule as it is physiologically overexpressed at this cell stage.

In apparent opposition to Woodcroft findings are those of Smith et al. [72]. It had been observed that loss of function-mutations of INK4A, which are very common in human lymphoid malignancies are seldom found in the subset of t (1; 19) chromosomal translocation-associated leukemias. This observation prompted Smith et al. to look for other oncogenes functionally redundant that could give rise to the same neoplastic phenotype. Using cDNA microarrays they identified Bmi-1 as a gene upregulated by E2a-Pbx1 in a human pre-B cell line (A2). In turn, downregulatioon of Ink4a-ARF by Bmi-1, confirmed in this cell line, offered an explanation to the negative correlation in pre-B cell leukemias between Ink4a-ARF loss of function mutations and t(1;19) chromosomal translocation.

Smith et al. performed myeloid replating assays using bone marrow c-Kit^+^ progenitors from wt or Bmi-1 deficient mice that were transduced with MSCV retrovirus encoding E2a-Pbx1 or chimeric oncoprotein E2a-Hlf. Numerous colonies exhibiting blast-like morphology were observed in third replating in all cases except in E2a-Pbx1 transduced Bmi-1^−/−^ cells which exhibited well differentiated colonies. Concurrently, p16 (and p19) expression was reduced in wt progenitors expressing E2a-Pbx1 consistent with downregulation by Bmi-1 overexpression. On the other hand, the normal differentiation observed in E2a-Pbx1 infected-Bmi-1 deficient progenitors suggests that transformation is mediated by Ink4a-ARF silencing induced by Bmi-1 expression.

The divergences between these two reports can be reconciled by taking into consideration the different experimental procedures used: a pre-B cell line that revealed Bmi-1 upregulation and a myeloid replating assay using c-Kit^+^ purified progenitors in Smith’s paper. Neoplastic colonies were identified simply as blasts but Bmi-1 absence and consequent p16, p19 expression could be sufficient to prevent transformation in a replating assay. Instead, Woodcroft et al. used lethally irradiated mice transplanted with lin^−^ BM (bone marrow) cells or cultured fetal liver progenitors encoding E2a-Pbx1 that revealed a complete block in B-cell development and a partial block in myeloid differentiation whereas leukemia with a myeloid phenotype was seen after transplantiation of cultured fetal liver progenitors transduced with E2a-Pbx1. These experimental conditions led to a complete block in B lymphoid differentiation and consequently a myeloid leukemia. In humans the t (1;19) translocation is thought to take place in committed B-lymphoid progenitors, thus later than the CLP stage where a complete E2a-Pbx1 induced block occurs. It can be surmised, as Woodcroft et al. did, that the neoplastic phenotype is determined by the developmental stage of the cell into which the oncoprotein is introduced. The two reports identified two different oncogenes as mediators of leukemogenesis: Hoxa9 and Bim-1. An elevated expression of either Hoxa9 or Bmi-1 was detected by each of them, although the overxpression of Hoxa9 was much higher than that of Bmi-1. Both these molecules have shown oncogenic effects in connection with myeloid and lymphoid lineages, respectively. However, the oncogenic effect of Bmi-1, as detected in myeloid replating assays, was revealed by the normal differentiation, despite E2a-Pbx1 introduction, of Bmi-1 null cells where the expression of Ink4a-ARF continuous to be present given the absence of Bmi-1, therefore preventing deregulated growth.

The oncogenic effect attributed to INK4-ARF loss of function mutation, most likely does not involve HSCs because Ink4a-ARF is physiologically silenced in HSCs as Bmi-1 expression is typically high in HSCs while decreasing with differentiation [73]. Furthermore, it has been shown, in single cell assays, that Bmi-1^−/−^ CD34^−^ KSL HSCs, that is LT-HSCs, undergo the first cell division in a fashion similar to that of wt control and showed no detectable apoptotic cell death, although total Bmi-1^−/−^ BM cells presented a slight but significant increase in apoptosis. Although defective self-renewal had been attributed to derepression of p16 and p19 in Bmi-1^−/−^ HSCs it was later shown that deficiency of these genes only partially reversed the self-renewal defect [73]. In fact, the impairment of self-renewal by derepression of p16, p19 is probably caused by the effect of these molecules on progenitors, not on HSCs where these repressor molecules appear to be ineffectual. In stem cells the INK4-ARF loci is most likely saturated by abundant Bmi-1 which silences expression of p16 and p19. Lack of Bmi-1 should result in derepression of these molecules, nevertheless suppression of HSC proliferation was not observed [73]. The concept that cell cycle control is fundamentally different in HSCs and post-stem cells and that this is determined, at least in part, by a molecule responsible for maintenance of stemness is an attractive hypothesis which could be at the core of the independent regulation of telomeres in stem and somatic cells and the resistance of stem cells to malignant transformation. It has been demonstrated that Bmi-1 determines the proliferative capacity of normal and leukemic stem cells [74]. In parallel, Bmi-1 overexpression or loss of function mutation of INK4a-ARF can induce leukemogenesis. The precedent experiments suggest that these tumorigenic effects cannot take place at the level of the stem cell. However, in post-stem cells upregulation of Bmi-1 could induce tumorigenesis either through silencing of Ink4a-ARF locus or by direct induction of telomerase [75], thus inducing stemness in the context of a partially differentiated cell.

### C/EBPα

C/EBPα is one of the genes expressed in HSCs. Nevertheless C/EBPα inactivating mutations or reduced expression are mainly linked to myeloid leukemia (AML). Unsurprisingly as it is an essential factor for myeloid differentiation, C/EBPα knockout (KO) mice undergo a complete block of neutrophilic development at the common myeloid progenitor stage [76]. However, a complete deletion of C/EBPα does not result in myeloid leukemia in mice as it blocks formation of granulocyte-macrophage progenitors (GMPs) and myeloid commitment, which indicates that some retention of residual myeloid function is necessary for myeloid leukemia generation. This is in line with the observation that partial developmental arrest rather than a complete block precedes leukemogenesis (PU.1 hypomorphic mice which express 20% of wild type PU.1 levels, develop a lethal AML, whereas conditional and non-conditional PU.1 knockout (KO) mice do not) [77]. The absence of myeloid leukemia induction in C/EBPα null mice has allowed other and earlier developmental defects caused by C/EBPα deficiency take center stage and let themselves for study. Thus,Wagner et al. [77] using fetal liver hematopoietic cells from C/EBPα wild type (WT), or heterozygous mice transduced with bcr/abl-GFP found that the expression of bcr-abl in these mice induced a myeloid leukemia but this outcome was not seen in C/EBPα null cells. The peripheral blood of C/EBPα null mice contained numerous erythroid precursors with normoblasts and erythroblasts comprising two-thirds of nucleated cells whereas, as expected, elevated granulocyte counts were present in the other groups of mice. Secondary transplanted mice with these immature erythroid precursors developed a malignant erythroleukemia. The immature erythroid cells of C/EBPα null mice showed elevated expression of Gata-1 and the stem cell leukemia gene (SCL). Id1 was also identified as a target of C/EBPα important for neutrophilic differentiation. This case could be presented as one of leukemia associated to a complete block in the myeloid lineage. However, it must be noticed that the erythroleukemia develops under concomitant expression of bcr-abl oncogene. Likewise, in the K562αER erythroleukemic line which expresses bcr-abl and Gata-1 but no C/EBPα, myeloid differentiation was induced by enforced expression of C/EBPα accompanied by decrease Gata-1 expression. Thus, lack of C/EBPα is associated to a differentiation block (in this case a complete block) with the tumor phenotype corresponding to an earlier stage of development. The erythroleukemic cells expressed bcr-abl. On the other hand HSC exhaustion which is most probably accompanied by telomere erosion is one outcome of C/EBPα deletion [76]. The signaling pathways leading to HSC exhaustion are not well known but the level of Id1 which is a target of C/EBPα should decrease in parallel to C/EBPα whereas E2A level that correlates inversely with Id1, could promote HSC entry into cycle and in the differentiation pathway. Thus, it appears that C/EBPα deficient HSC are increasingly mobilized in the presence of blocked myelopoiesis and may have undergone accelerated telomere erosion before engaging the megakaryocyte-erythrocyte progenitor (MEP) route of development.

Like C/EBPα, PU.1 is expressed in HSCs and is upregulated in GMPs during granulocyte and macrophage development and downregulated in MEPs. The erythroleukemia cell line MEL harbors the friend virus integrated in a crucial driver of PU.1 expression, the upstream regulatory element (URE) of the Spi-1 gene (PU.1). This viral integration cause aberrant expression of PU.1 in the erythroid lineage in conjunction with Gata-1, leading to arrested differentiation which appears to be the result of the contradictory signals mediated by PU.1 that blocks erythroid differentiation and Gata-1 that stimulates it [78]. Deregulation of this network may result in erythroleukemia [78]. Again, in this case leukemogenesis seems to be associated to a complete differentiation block but **more probably** the arrest is determined by the stoichiometry between PU.1 and Gata-1. Therefore, the contradictory signals delivered by GATA-1 and PU.1 pushing in two opposite directions may result in a situation akin to a partial blockade. Interestingly, the leukemic phenotype of cells blocked at the proerythroblast stage of differentiation can be reversed when these cells are treated with different agents such as HMBA, DMSO or additional Gata-1 that help to overcome the block and resume differentiation. A similar reversal of the tumor phenotype is observed in the treatment of acute promyelocytic leukemia (APL) with agents that promote differentiation like all-trans-retinoic acid (ATRA) [78]. Cells arrested at a given stage may self-renew indefinitely and undergo either telomerase overexpression or telomere erosion. However, tumor inhibition achieved by differentiating agents may suggest that the leukemic state was maintained through telomerase overexpression.

### CEBPA mutations with partial loss of function

Around 90% of these leukemias harbor 1 C/EBPα allele with a C-terminal mutation containing in frame insertion/deletions within the DNA binding domain (K allele, p42) and another allele with altered N-terminal region (L allele or p30 isoform). As stated above, a complete deletion of CEBPA does not lead to leukemia in the mouse although it blocks the formation of GMPs and myeloid commitment. Instead CEBPA heterozygous mutations impair E2F/p107 repression by CEBPA [36] and increase myeloid progenitor proliferation. By generating fetal liver cells from K/K, K/L, L/L and +/+ mice, an expansion of LSK compartment, most significant in the ST-HSC (CD150- Flt3-) and LMPP (CD150- Flt3+) was observed in K/L and K/K but not in L/L mice compared to controls. In addition, the expression of quiescent-associated genes was significantly decreased in K/K and K/L but not in L/L populations. Leukemia development was faster in K/L mutants but all genotypes were equally susceptible to leukemia. K/L and L/L leukemias were characterized as granulocytic with maturation (FAB M2). Only 25% of K/K leukemias qualified for myeloid with maturation with the remaining exhibiting a more immature phenotype qualifying for leukemia with erythroid lineage involvement. GMP were virtually absent in this leukemias suggesting a persistent block that prevented generation of committed myeloid cells. The authors suggested that the immature phenotype was responsible for the delayed kinetics. In agreement with the phenotypic features, myeloid genes were depleted from K/K and K/L LSK cells but much less from L/L cells. However, by separating the leukemic population from each genotype into an HSC-containing fraction (Sca-1^+^), a myeloid-progenitor fraction (Sca-1^−^, Mac-1^lo/+^ c-Kit+) and a differentiated fraction (Sca-1^−^ Mac-1^hi^ c-Kit^lo/−^, it was shown that only the myeloid-progenitor fraction gave rise to leukemia. The HSC-containing fraction yielded long-term multilineage engraftment with no leukemia observed within 4 months [36].

### C/EBPα involvement in other oncogenic signaling pathways

Within the multipotent progenitor compartment FLT3 is produced in cells skewed to lymphomyeloid but limited erythromyeloid lineage [73]. These cells express a set of lymphoid lineage genes such as Rag1, Tdt and sterile Ig transcripts. In the absence of E2A(TCF3), IKZF1, MYB or PU.1, expression of FLT3 as well as other lymphoid lineage genes is reduced. Flt3 mutations in leukemia are activated mutations which suggest that the mutation must hit a cell of origin located at or downstream of LMPP stage [73]. Furthermore, Flt3 overexpression promotes leukemization at least in part by inducing arrested maturation through downregulation of C/EBPα [79]. Myeloid leukemia driven by Flt3 activation appears to require the differentiation block mediated by Flt3 inhibition of C/EBPα. Still, a mechanism that may reverse differentiation may also be invoked here. It has been shown that C/EBPα reprograms B cell precursors to macrophages [80]. This suggests that inhibition of C/EBPα by Flt3 could reverse this pathway in the direction from macrophages to lymphoid cells. On the other hand, in most cases Flt3 internal tandem duplication (Flt3ITD) mutations cooperate with other mutations leading to differentiation blocks.

### Maturation arrest in chromosomal translocation-associated leukemias

A great fraction of myeloid leukemias of infancy are associated to chromosomal translocations that generate fusion proteins which combine different domains of the two genes joined by the translocation. The consequences generated are twofold: halting of the normal course of cellular differentiation (class II mutation) that was induced by the normal gene product and stimulation of cell proliferation (class I mutation) or, in other words, stimulation of cell proliferation at an inappropriate stage of development. Biological effects caused by the chromosomal translocated products AML-ETO, promyelocytic leukemia-retinoic acid receptor (PML-RARα) and PLZF-RARα all include partial differentiation arrest and enhanced cell proliferation apparently exceeding developmental stage boundaries [81–83]. AML-ETO acts mainly as a dominant negative mutation of the normal AML1 gene by recruiting corepressor molecules such as N-Cor, mSin3A, SMRT and histone deacetylases to AML1 DNA binding sites. Developmental block associated to AML-ETO has been shown to depend on the ability of AML-ETO to suppress C/EBPα expression [84] but it may also block differentiation by suppression of granulocyte-macrophage-colony-stimulating factor (GM-CSF) [85]. PML-RARα and PLZF-RARα induce a block in hematopoietic differentiation by recruiting repressor complexes to retinoic receptor target genes. Arrested maturation occurs in a rather advanced promyelocyte stage and can be relieved by ATRA in the case of PML-RARα. However, PLZF-RARα recruits polycomb repressive complex 1 (PRC1) which is insensitive to ATRA [86]. The ability of all these translocation products (AML-ETO, PML-RARα and PLZF-RARα) to activate Wnt signaling through induction of plakoglobin (γ-catenin) which blocks β-catenin degradation and activates TCF, LEF and their target gene c-myc contributes to enhanced cell proliferation and preservation of immature features [87]. As stated above, c-myc can activate TERT expression. Moreover, c-myc could be responsible for TERT expression independently of telomere damage. Activation of this pathway probably underlies the AML-ETO induced expansion of human HSCs and/or progenitors measured as cobblestone area-forming cells in a report by Mulloy et al. [88]. The expansion of HSC subsides after a few rounds whereas myeloid cells proliferate continuously [88]. Undoubtedly, the three fusion proteins qualify as both type 1 and type2 oncogenes.

Repression of C/EBPα has also been proposed to be involved in the pathogenesis of PML-RARα [86]. All these studies suggest that even if the fusion protein is present in HSCs, transformation takes place in downstream cells. The PML-RARA fusion protein associated to the M3 subtype of AML is not present in the CD34+ CD38- HSC population but the AML-ETO fusion protein is detected in normal bone marrow cells as well as in leukemic blasts.

Evidently, arrested maturation caused by different transcription factors (TFs) or at different stages of development may vary in their ability to induce the TERT oncogenic pathway. This pathway may be triggered, to our knowledge, by telomere damage, c-myc overexpression or Bmi- [75] and can be negatively modulated by p53.

Transformation induced by either MLL fusion proteins or MLL dimerization appears constantly associated to partial myeloid differentiation arrest and Hoxa9, Meis1 overexpression [89, 90]. These proteins activate an HSC-self-renewal program in downstream cells. Interestingly, Hoxa9 and Meis1 are upregulated in advance of the full self-renewal signature [91]**.**

The A cluster Hox genes, including Hoxa7, Hoxa9 and Meis 1 are, under physiological conditions, only expressed in c-Kit+,Thy1^lo^ Lin1^lo/−^,Sca1+ rhodamine 123Rh^lo^ hematopoietic stem cells (HSCs) and their expression is rapidly downregulated in more differentiated multipotential, common lymphoid and myeloid progenitors [92] However, dimerized MLL and MLL-AF9 (or other fusion proteins) show persistent expression of Hoxa7, Hoxa9 and Meis1 [92]. Expression of these factors beyond physiological boundaries of expression might cause replication stress and telomere erosion. Nevertheless, the fusion protein MLL-GAS7 has been reported to induce transformation in the absence of Hoxa9 although penetrance is reduced to 50% [93]. The role of Hoxa9 and Meis1 has been repeatedly confirmed in other MLL translocations [94]. Cord blood (CB) cells infected with a retrovirus expressing MLL-AF9 differentiated normally in in vitro cultures shortly after transduction but became immortal between weeks 5 and 19 of in vitro culture. Interestingly, hTERT activity was detected at early and late times in vitro suggesting that TERT may be induced also independently of telomere erosion [95]. In vitro growth of the MLL-AF9 transduced human CB cells was strictly dependent on the Flt3 ligand (FLT3L) and this could not be overcome by the combined use of stem cell factor (SCF), GM-CSF and IL-3 [95]. This clearly indicates that both the cancer stem cell and its normal precursor cell expressed FLT3 which marks these cells as located downstream of the HSC. On the other hand, Somervaille et al. demonstrated in a mouse model of MLL-AF9 that leukemia stem cells express mature myeloid lineage specific genes and account for a great proportion of tumor cells (25–30%) [96]. Schreiner et al. [97] showed that MLL-ENL caused a reversible block in myelomonocytic differentiation that was dependent on c-myc. MLL-ENL transduced bone marrow cells (BM) in culture were partially arrested as immature myeloid cells displaying an intermediate surface level of GR-1 and Mac-1 (CD11b). However, addition of G-CSF (granulocyte colony stimulating factor) induced terminal differentiation as well as proliferation arrest. A dominant negative myc mutant prevented the block caused by MLL-ENL and precluded transformation whereas MLL-ENL and c-myc co-transduced cells yielded higher number of colonies in third round replating and excess Myc rendered arrested maturation irreversible. On the other hand, some MLL-ENL transduced clones became spontaneously insensitive to the differentiating effect of enhancer α (Eα) which regulates Vα-Jα recombination after prolonged culture (> 4 months). The fact that maintenance of a myc-dependent block results in irreversible transformation suggests that myc induction of telomerase may require cooperation of some sort to initiate transformation or must elicit accumulation of TERT expression. Interestingly, Myc also arrested cells at an earlier stage (Mac-1 negative). Another factor which, like c-myc, can induce telomerase expression and therefore drive the TERT-TGFβ pathway is Bmi-1 [98]. Harada et al. have shown that Bmi-1 overexpression could collaborate with RUNX1/AML D171N mutant (the most frequent AML mutation) in development of a high-risk myelodysplastic syndrome [98]. The claim that HSCs are direct targets of leukemization in the MLL-ENL model has been made by Cozzio et [99]. These authors sorted murine hematopoietic cells from HSC, common myeloid progenitors (CMP), granulocyte-macrophage progenitors (GMP) and mehakaryocyte-erythroid progenitors (MEP) compartments and transduced them with retrovirus containing MLL-ENL cDNA. Upon culture in methylcellulose numerous colonies displaying blastlike morphology were present in third round plating of the HSC, CMP and GMP compartments. Culture of these cells in liquid media generated immortal, factor-dependent cell lines. Blast-like cells from all origins exhibited an identical immunophenotype: Sca-1 -, c-Kit^lo/int^, CD34+, FcϒR high, Gr-1 low, Mac-1 high. Therefore, immortal cells from all compartment origins seemed to be arrested at identical stage of differentiation. In parallel, the ability to generate leukemia in vivo was tested by transplanting MLL-ENL transduced HSC, CMP or GMP cells into syngeneic irradiated recipients. HSC (103 cells, *n* = 23), CMP (104 cells, *n* = 17), GMP (104 cells, *n* = 31). Donor populations were cultured in IMDM containing 20–50% virus stock, 10% FCS, 2ME, polybrene, SCF, Flt3L and IL-11 for 12–18 h and mixed with C57Bl/6. Ly5.1 congenic BM cells before transplantation. A similar onset and incidence of leukemia was observed in the recipients of cells from the three different compartments. The transformation efficiency was estimated to be HSC > CMP > GMP. Leukemic cells from the distinct origins displayed identical phenotype: Sca-1 -, c-Kit^lo/int^, CD34+/high, FcϒR high, Gr-1^lo^, Mac-1^hi^. This work unequivocally established that committed cells can be the target cells of origin of leukemias. However, the claim that HSC have a stronger or even a similar propensity to develop leukemia can be objected because all tumor cells independent of the transduced compartment are arrested at the same differentiation stage implying again that HSC must engage in hematopoiesis in order to become transformed.

Other homeobox-genes that are responsible for a subset of T-ALL are the TLX1 and TLX3 genes. TLX1 and TLX3 are normally expressed only during embryogenesis but their expression can be activated in postnatal life as a consequence of a chromosomal translocation. Then, homeodomain-mediated binding of TLX1 or TLX3 to transcription factor ETS1 results in repression of the enhancer α (Eα) which regulates Vα-Jα recombination, leading to arrested maturation at this stage. As the TCRα recombination takes place at the double positive (DP) stage of T cell development following recombination of TCRγ, δ and β, the phenotype of the arrested thymocytes corresponds to a previous stage of development: a double negative (DN) stage that may have rearranged TCR γ, δ or β genes but without surface TCRαβ. This is the phenotype shared by TLX1 and TLX3 associated leukemias, implying that the cell of origin of this T-ALL subgroup is a cell that was blocked at that precise stage of differentiation [100]. An interesting, additional feature revealed by this work was the observation that, upon release of the block by short hairpine RNA (shRNA) targeting TLX, the arrested lymphocytes could initiate differentiation but this was followed by cell death by apoptosis. A plausible explanation for this fate is that the enhanced or sustained lymphocyte proliferation during the block may have resulted in telomere attrition albeit this may have been partially compensated by telomerase expression. However, telomerase expression may have declined after reprisal of differentiation and damaged telomeres should reveal inadequate to sustain further cell growth.

### Developmental anomalies that can induce leukemia independently of arrested maturation

Some animal models have shown that leukemia can be induced through anomalous activation of cell proliferation at an inappropriate stage of developmental. Apparently oncogenesis in those models does not involve developmental blocks but it may require replication stress and subsequent telomere erosion.

Notch1 signaling controls early development of progenitors into the T cell lineage while inhibiting B-cell differentiation. Early thymic progenitors (ETPs) with the phenotype CD44+ CD25- (DN1 stage) give rise to dendritic cells, NK cells, and T lineage committed CD44 + CD25+ cells (DN2 stage). Lineage commitment is followed by VβDβJβ recombination which in combination with a surrogate light chain and other molecules such as CD3 chains forms the pre-TCR complex. Completion of the pre-TCR divides the DN3 stage into DN3a and DN3b. Pre-TCR signaling is needed for differentiation of CD44- CD25+ (DN3) into DN4 and DP cells. Disruption of pre-TCR arrests T cell development at the CD44- CD25+ stage. Rag null cells cannot re-arrange TCR genes and lack pre-TCR. Therefore, they fail to form DP T cells and arrest at CD44- CD25+ (DN3 stage). When Rag null are transduced with Notch ligand ICN1 do not generate DP. If, in addition, they are transduced with a TCRβ transgene they form DPs and rapidly expand indicating that preTCR signals complement Notch to develop DPs. ICN1-induced BM DPs from both WT and Rag null x TCRβ rapidly expand. In contrast, ICN1-induced Rag null did not, suggesting that pre-TCR signals are required for the proliferation burst that accompanies thymocyte differentiation. Mice repopulated with ICN1-transduced WT HSCs generated extrathymic DP T cells within 3 weeks. In contrast, mice repopulated with ICN1-transduced Rag-2−/− did not generate DP T cells. The ICN1-induced CD44 + CD25+ and CD44-CD25+ BM populations constituted 1–4% of all BM cells. These findings are consistent with a model where Notch commits lymphoid precursors to the T lineage but pre-TCR signaling is required for the proliferative burst that accompanies thymocyte differentiation.

Recipients of ICN1-transduced HSCs from Rag null mice remained alive for > 1 year after transfer. In contrast all mice receiving ICN1-transduced BM cells from Rag null mice expressing a TCRβ transgene developed T cell leukemia between 9 and 11 weeks after transfer. Thus, ICN1-mediated transformation of T cell progenitors required expression of a TCRβ chain and development of CD4+ CD8+ T cells [101]. This raises the question whether leukemia induction has been promoted by the further differentiation of T cells into a DP stage or by the proliferative burst resulting from a functional pre-TCR plus Notch expression. However, it is known that under physiological conditions thymocyte proliferation ceases shortly after the cells reach the DP stage and Notch signaling fails to influence thymocyte development following β selection [102]. These observations suggest that the likely event underlying leukemia development is the occurrence of Notch signaling in DP cells, a developmental stage not normally subjected to this signaling. This amounts to a Notch input beyond its physiological temporal range which might lead to replication stress and increased telomere erosion.

PTEN deficiency may contribute to oncogenesis through different routes. As an inducer of genetic instability it may impair the accuracy of the recombination of TCR and IgH genes. In a mouse model involving conditional deletion of Pten in 40% of fetal liver HSCs by the expression of VE-cadherin-Cre, Pten deficiency leads to a t(14;15) chromosomal translocation involving the Tcra and c-myc loci and subsequent T-ALL formation similar to a t(8;14) associated with a subset of human T-ALL. The driving oncogenic event in this leukemia is the activation of c-myc gene whose expression under the control of the Tcra promoter occurs at an inappropriate stage of lymphocyte development. Activation of β-catenin is detected in some cases but is not an immediate event following translocation. β-catenin can increase the penetrance and rate of leukemogenesis but is not required for leukemia development. As V(D) J recombination takes place at the DP stage of lymphocyte development, this must be the cell stage of origin of leukemic transformation in spite of Pten deletion occurring in HSCs. In fact, the leukemia stem cell (LSC) was identified as a CD3+ c-Kit^mid^ and c-myc overexpression was detected in LSCs and CD3+ but not in HSCs or CD3- cells [45]. According to the former findings leukemization occurs at a certain stage of lymphocyte development which at least in this case is not an HSC even if Pten deletion has taken place in a HSC. The developmental stage of the cell of origin dictates the leukemia phenotype. T-ALL associated to Pten deficiency may develop independently of a chromosomal translocation as long as activation of cell proliferation/differentiation occurs at an inappropriate stage of lymphocyte development. By creating doubly deficient Pten; Rag mice, it was demonstrated that Rag-mediated double strand breaks (DSBs) enabled the generation of Tcra-c-myc translocation. In the absence of Rag mediated recombination t(14;15) associated leukemia did not develop. It was observed that BM transplantation of double Pten; Rag null mice did not give rise to leukemia during 5 months after BM transplantation but recipients developed an enlarged thymus and, in the absence of antigen rearrangement leukemia could not be ruled out. Rag null thymocytes accumulate at the DN3 stage because of β-selection blockade but did not undergo leukemization which suggested that blockade by itself is insufficient to induce leukemia. Pten loss enables Rag null thymocytes to bypass β-selection and differentiate to DP thymocytes [103]. This is the stage susceptible to c-myc driven leukemization caused by t (14;15). Other researchers showed that Pten−/− Rag1−/− could undergo leukemization in the absence of t(14;15). By crossing CD4-Cre x Pten^fl/fl^mice onto the recombination deficient Rag1−/− Liu et al. [104] conditionally deleted Pten in the DP stage using TCR transgenic (OT-II-TCR) in order to bypass the β-selection blockade and therefore observe the effect of Pten at this stage independently of chromosomal translocation. These mice developed CD4+ CD8+ lymphomas limited to the thymus expressing low levels of TCRβ and no t(14;15) clonal translocation. It was found that c-myc overexpression was also the main driving force in this leukemia although in this case c-myc was under the control of the Notch pathway [104]. Former experiments had already revealed that c-myc is a downstream target of the Notch pathway [105].

On the other hand it is known that the Notch pathway is active during T cell development but becomes downregulated after β- selection. Notch expression later than this checkpoint may promote tumorigenesis as stated above and [106]. This is in line with c-myc upregulation induced by Notch. DP thymocytes normally undergo differentiation arrest but enforced proliferation caused by c-myc or any other factor at this stage may be a physiologically disturbing event with the potential to undermine telomere function. At this point, it may be fitting to point out that a partial blockade may be also involved in transformation. As a matter of fact, Notch does not induce ATL (acute T leukemia) in pre-TCR deficient mice, probably because pre-TCR deficiency leads to a complete differentiation block. Nevertheless, even transient activation of pre-TCR signaling with antibodies specific for CD3e restores the ability of Notch to induce TALL [106]. Retrospectively, it could be surmised that the enlarged thymus observed by Guo et al. [103] contained a lymphoma since Pten loss without collaboration from OT-II-TCR can bypass β-selection and attain the DP stage Other interesting finding reported in Guo paper [103] was that rapamycin, an inhibitor of mTOR (downstream target of PI3K/AKT pathway upregulated by Pten deficiency), could not reduce (and therefore inactivate) P-S6 level of the LSC subpopulation whereas it reduced P-S6 level of CD3+ blasts. This finding explained that rapamycin treatment decreased tumor bulk which is comprised predominantly by CD3+ blasts but could not affect LSCs resulting in an increase ratio of LSCs to blasts. Some conclusions can be drawn from these findings:

1. It is not expected that HSC should be the direct ancestor of the LIC (leukemia initiating cell) that arises after a chromosomal translocation occurring during TCR recombination but, nothing a priory, precludes that HSC would be the cell of origin of Notch1/c-myc induced leukemia in Pten deficiency. However, this is not observed in spite of the ability of Notch pathway to induce proliferation of HSCs. Similarly, activating mutations of Wnt pathway are not typically identified as driver oncogenic mutations in leukemia even though Wnt3A has been shown to cause up to a 300-fold expansion of LT-HSCs [7].
2. Induction of cell proliferation at the wrong developmental stage rather than enhanced cell proliferation alone may promote tumorigenesis. The contribution of a partial developmental block could also be involved in these cases.
3. the leukemic phenotype coincides with the phenotype imparted by the developmental stage of the cell of origin.
4. In a given leukemia some mutations act as oncogenic drivers and others are merely contributory (in these T-ALL, c-myc and β-catenin).
5. Resistance of the LSC to the inhibitory effect of rapamycin coupled to the ability of this molecule to inhibit the more differentiated CD3+ population suggests that the cell of origin of leukemia, which belongs in a CD3+ stage of development, must have acquired resistance to rapamycin as a consequence of leukemic transformation.

### Early T cell leukemias (ETP-ALL)

ETP-ALL (early thymic precursor acute lymphoblastic leukemia) was recognized as a novel entity by Coustan-Smith et al. [107]. This leukemia subtype accounts for about 10% of acute T-lymphoblastic leukemia. It is identified by an immature immunophenotype characterized by absent CD1a and CD8 expression, week CD5, cytoplasmic or surface CD3, CD7 and a gene expression profile including T-lineage and stem-cell and myeloid transcripts. Some transcripts like LYL1, ERG, LMO1 were common to ETP-ALL and T-ALL. One rearranged TCR gene at least was identified in 8 of 9 cases studied. These features point unambiguously to an origin in a thymic or prethymic early progenitor. Zhang et al. [108] studied the genetic profile of ETP-ALL by whole genome sequencing of 12 ETP-ALL cases. The mutational spectrum included histone modifying genes, cytokine receptors and inactivating mutations in genes regulating development. In addition to genes known to be mutated in T-ALL like NRAS, NOTCH1, JAK1, IL7R, there were mutations in genes involved in early T cell development such as GATA-3, EP300 and RUNX1. Transcription of GATA-3 at low level has been detected in HSCs but transcription gradually increases between ETP and DN3 stages. Several experiments suggest that GATA-3 is dispensable for the development of pre-thymic T-cell progenitors but is certainly required for the development of early thymus progenitors (ETPs) [109]. An animal model supports origin downstream of pre-thymic cells [110].

In summary, although Zhang et al. inferred that ETP-ALL is stem cell leukemia, under the criteria adopted in this paper, ETP-ALL arises in a relatively committed cell downstream of LMPP and pre-thymic stages. More significant, in Zhang’s report telomere erosion was the most constant and unifying feature.

#### Corollary

The conditions described above encompass genetic mutations that cause arrested maturation (class II mutation) as well as others inducing cell proliferation at the wrong stage of development which could be considered a subclass of class I mutations. Both of them might lead to replication stress and subsequent telomere damage, although of all the cases described, there is information on telomere length only in the case of very early T cell leukemias where telomere length was measured, and shown to be reduced as compared to non-neoplastic cells. This reinforces data coming from a report showing evidence for a pre-existing telomere deficit in non-clonal hematopoietic stem cells in acute myeloid leukemia [10]. However, as data on telomere erosion is not available, in most other cases, the involvement of telomere pathways on their associated leukemias remains hypothetical. However, the consistent association of hematopoietic developmental arrest with cancer transformation suggests that the multiplicity of transcription factors responsible for arrested maturation converge on a common step which is essential for transformation. This common step could be identical to the shared telomere damage and signals downstream of it. After discarding severe telomere attrition on the grounds stated above for acute leukemias, other telomere related mechanisms are plausible. Blocked differentiation may trigger indefinite self-renewal which entails either telomerase overexpression or telomere loss and both of these outcomes may promote tumorigenesis. Telomerase was shown to be tumorigenic when transfection of hTERT immortalized and converted human mammary epithelial cells to a stem-like state [38]. Another mechanism that could be involved here would be similar to that underlying the findings discussed above of TASP and SASP where association of moderate telomere damage with transformation was found. Finally, a mechanism can be invoked which is triggered from a different type of telomere alteration, shown to be tumorigenic. Recently, it was reported that the apoptotic endonuclease G (Endo G) affects alternative splicing of hTERT gene by reduction of α + β + hTERT variants and increasing expression of α + variant. Other apoptotic endonucleases induce, like Endo G, cell death but are unable to modulate hTERT splicing. The hTERT variant (as induced by Endo G) does not show telomerase activity but can induce immortalization [111]. Overexpression of Endo G in sorted CD4+ T cells downregulated the active full length hTERT variant and upregulated the α + variant. This led to massive apoptosis of cultured T cells but also to the emergence of solitary transformed cells with blast morphology, the immunophenotype of a T cell lymphoma and the ability to form tumors and cause death in transplanted mice. The great diversity of driving ocogenic events discussed may share a final common oncogenic mechanism that lies in some form of telomere complex alteration. This parallels the in vivo emergence of cancer stem cells in vitro as seen in the phenomenon known as crisis, as postulated in a former paper [15].

As this survey shows most leukemias originate from post-stem cells suggesting that cells may have lost a protective mechanism associated to stem cells This is consistent with the concept that some form of telomere damage is necessary for malignant transformation.

As pointed out above Rap1 may connect signals arising from telomere alteration to several routes with oncogenic potential. It can activate NF-κB, a marker of leukemic stem cells absent in normal hematopoietic stem cells. NF-κB activation correlates with DOT1L activation which has been shown to be required for development of MLL-rearranged leukemias. Paradoxically, DOT1L induces transformation by upregulating HOXA9 and MEIS1 while in parallel it can impair somatic cell reprogramming (at least in ageing) [112, 113]. These apparently opposing effects of DOT1L appear contradictory. It is possible that re-expression of telomerase collaborates with DOT1L in order to overcome the barrier to transformation associated to impaired reprogramming and/or that an incomplete reprogramming induced by DOT1L is essential to the cancerous state. We have seen above that Rap1 can also promote Foxo3a inactivation and that positive IKK staining, p-AKT and cytoplasmic Foxo3a correlated in 90 out of 130 human primary breast specimens.

### Primary genetic derangement of the TERT-TGFβ oncogenic pathway

In the preceding pages the TERT-TGFβ pathway has been considered an oncogenic pathway exclusively involved in tumorigenesis of post-stem cells in which it would be triggered, at least in most cases, secondarily to replication stress and subsequent telomere damage. However, there is the case of a primary derangement of this pathway in the stem cell disorder Beckwith-Wiedemann syndrome. This genetic disease is associated with an 800-fold increased risk of childhood neoplasms with multiple tumors arising in different organs or independently in the same organ. Overexpression of telomerase reverse transcriptase (TERT), defective TGFβ signaling with epigenetic silencing of β2- spectrin, a SMAD adaptor for TGFβ signaling and impaired feedback repression of TERT by TGFβ are involved in the etiology of this disease [114]. Contrary to other situations examined in this paper where deregulation of TERT-TGFβ occurs in downstream poststem cells, in B-W syndrome deregulation is primary and may be expressed in stem cells. Thus, after all stem cells might be no less susceptible to malignant transformation than cells in downstream developmental stages (although they would still be protected by the exceptional occurrence of this mechanism in the stem cell compartment). This question will be examined in the following section.

### Section 2

#### A specific resistance of stem cells to neoplastic development

The preceding observations argue strongly for the prominent role played by the telomere attrition cascade in leukemogenesis. However, this question is inextricably linked with that of the stem cell origin of cancer and therefore the rarity of true stem cell tumors could alternatively be attributed to a specific resistance of stem cells to carcinogenesis. Here, I gather experimental and clinical evidence for the concurrent existence of a specific resistance of stem cells to malignant transformation. Whether promotion of leukemogenesis is associated to partial versus complete developmental blocks is not a settled issue: if only partial blocks can lead to leukemization, the absence/rarity of stem cell leukemias could be expected since a partial, immediate post-stem block would give rise to leukemization of the few HSC that passed the block and reached the stage of early progenitors. Still, it would suggest that leukemization requires exit from the HSC compartment and is accelerated by partial maturation arrest. For instance, heterozygote or homozygote deletion of E2A cannot induce a complete block before CLP stage since other E proteins (HEB, E2–2) can generate at least partial lymphocyte differentiation. However, complete arrested maturation at this primitive stage induced by inhibition of all E proteins mediated by Ids as it has been shown by generating induced leukocyte stem cells [70] does not lead to leukemization after several months when arrested at this early stage. Thus, it seems that a complete developmental block unassociated to proliferation cannot induce transformation. Blocking development at the immediate post stem stage concurrently with induction of stem cell proliferation should unveil the resistance of stem cells to transformation.

Gata 2 plays an important role in the self-renewal and maintenance of HSC. Gata 2 expression is under the control of Evi1 which is predominantly expressed in embryos and adult bone marrow HSCs. Ectopic expression of Evi1 or Gata2 in Evi1−/− mice restores maintenance and proliferation of HSC [115]. Quiescence of HSC appears to require interaction of Tie2 (a downstream target of Evi1-Gata2) with its ligand Angiopoietin-2 (Ang-1). This promotes adhesion of stem cells to their niche. It has been shown that long-term repopulating HSCs (LTR-HSCs) that adhere to niche osteoblasts can be labeled by BrdU for relatively long periods [116]. Apart from reduction of the HSC compartment, further hematopoietic differentiation of Evi1 heterozygous is not grossly impaired except for a detrimental effect on the myeloid lineage that results in the absence of granulocytes in recipients of explants from Evi mutant mice [115]. This could be related to the ability of Gata 1 and Gata2 to interact with the myeloid lineage and B-cell regulator PU.1, which, in turn, may underlie the predisposition to myeloid malignancies associated to Gata2 mutation. On the other hand, Gata 2 hypomorphic mice show upregulated expression of cytokine receptors in myeloid progenitors and granulocytosis and monocytosis upon exposure to inflammatory cytokines [117]. In the human hematopoietic system, the main manifestations of Gata 2 deficiency are dependent on the HSC compartment failure which gives rise to a hypocellular bone marrow and multilineage dysplasia accompanied by subsequent cytopenias in all lineages. In a revision of 57 patients with Gata 2 deficiency, Spinner et al. [118] detected B cell, NK, monocyte and CD4-T and neutrophil cytopenias in 86, 82, 78, 51, and 47% of patients respectively. Pancytopenia was observed in the setting of MDS (myelodisplastic syndrome) and atypical megakaryocytes in 92% of cases sometimes without MDS; 14% of patients developed AML and CMML was diagnosed in 8%. Interestingly, in addition to hematopoietic malignancies, solid organ tumors were frequent, some of them related to viral infections such as HPV, but squamous carcinomas, mesenchymal tumors, breast cancers, adenocarcinoma of pancreas, renal cell carcinoma and an invasive desmoids tumor were described [118]. Similar findings have been reported by McReynolds and al [117] These authors also found a single case of pre-B-cell ALL and underlined the frequent cooperation with other gene mutations such as ASXL1 described in 29% of hematopoietic clones in one series [119]. Involvement of Gata2 in the maintenance of other tissue stem cells is quite probable. Therefore, it can be hypothesized that undermining of self-renewal and deficient maintenance of the stem cell compartment of different tissues is responsible for the generalized predisposition to tumor development associated to Gata 2 deficiency. The mechanism could be similar to that underlying the predisposition of atrophic conditions to cancer involving undermining of regenerative potential and possibly associated telomere deficiencies. In the hematopoietic system additional factors such as the specific role of Gata 2 in myeloid development and cooperating mutations such as mutation of ASXL1 gene may play an additional role especially in myeloid malignancies [119] As expected from their respective roles in hematopoiesis, Gata2 expression precedes Gata1 expression. Gata2 activates Gata1 which drives differentiation of HSCs towards the erythroid/megakaryocyte lineage. In the absence of Gata1 erythroid progenitors undergo apoptosis whereas megakaryocytes fail to undergo terminal differentiation and expand dramatically [120] Megakaryocyte proliferation promotes development of acute megakaryoblastic leukemia (AMKL) frequently associated with Down syndrome (DS). It is noteworthy that an accelerated loss of telomeres has been described in patients with Down syndrome [121]. Nearly all cases of AMKL and transient abnormal myelopoiesis (TAM) in children with DS contain acquired Gata 1 mutations [120]. The underlying cause of this association is unknown. However, telomere loss in DS may occur in the absence of Gata 1 mutation and has been considered to be the primary event leading to LT-HSC deficiency and leukemia predisposition [46, 122]. Other conditions linked to telomere dysfunction are also associated to HSC failure and leukemia predisposition such as dyskeratosis congenita (caused by TERC mutation) and a hereditable heterozygous mutation of TERT responsible for autosomal dominant aplastic anemia [46].

Other gene expressed at the initial stage of hematopoiesis is the stem cell leukemia gene (SCL/TAL1). In the adult, SCL levels are highest in HSCs. Although knockout models failed to demonstrate a specific requirement of SCL in the maintenance of HSC, human cells overexpressing SCL showed increased reconstitution ability and serial transplantation of cells with a single SCL allele was shown to be significantly impaired. Since SCL heterodimerizes and inhibits E2A/TCF3 it is likely that SCL helps to maintain HSC quiescence through E2A inhibition. During hematopoiesis, SCL is absolutely necessary for erythroid and megakaryocytic differentiation and in its absence B-cell differentiation is hindered after the CLP stage. However, the leukemogenic role of SCL is mainly associated to chromosomal translocations that juxtapose Tcrα or Tcrβ regulatory sequences to SCL with subsequent overexpression of SCL that drives DN3 proliferation. Since SCL levels are under physiological conditions undetectable in DN3 thymocytes it seems reasonable to hypothesize that leukemization may be promoted by inappropriate timing of cell proliferation rather than by proliferation itself and that this aberrant proliferation might determine telomere damage in a much greater degree than physiological cell proliferation [123].

Leukemogenesis driven by c-Kit is not known to be dependent on a preleukemic differentiation block. C-Kit is expressed on HSCs as well as in myeloid, erythroid, megakaryocytic and dendritic progenitors and mature mast cells. This implies that activating mutations of c-Kit can potentially induce leukemias originating at any of these developmental stages. It must be underlined that c-Kit is more strongly expressed in LSCs than in normal HSCs [2] According to this observation, it might be expected that constitutive activation of c-kit in the HSC compartment could generate leukemia with a stem cell phenotype. However, activating mutations of this receptor tyrosine kinase have been found associated to AML and, predominantly, mastocytosis but not stem cell leukemias [124].

#### Expression of the TERT-TGFβ pathway in HSC results in loss of quiescence and leukemogenesis apparently arising downstream of HSCs

Perhaps the most compelling findings supporting the concept of a direct origin of leukemia in HSCs are those reported by Passegué et al. [49]. In this report, similar to Beckwick-Wiedemann syndrome, leukemogenesis is driven by the TERT-TGFβ signaling pathway which is active in HSCs and GMPs. They found that AP-1, a heterodimeric complex of the Jun (c-Jun, JunB, or JunD) and Fos (c-Fos, FosB, Fra-1 or Fra-2) family proteins, represses TERT activity and that repression of TERT leads to reduced numbers of HSCs as well as reduced CMPs and CLPs. By reintroduction of JunB expression under the ubiquitin promoter (Ubi-JunB) in JunB−/− mice (junB−/− −Ubi-JunB) embryonic lethality of JunB−/− mice was rescued but these mice developed a myeloproliferative disease. Interestingly, the HSC and GMP compartments were expanded before the appearance of MPD without a concomitant increase in the intermediate compartments MPP or LMPP. By the time of appearance of MPD (between 6 and 9 months), JunB mRNA expression was dramatically reduced in all hematopoietic cells. MPD could be transferred to sublethally irradiated immunocompromised recipients by LT-HSC isolated from 6 to 9 month old junB−/−Ubi-junB mice but not from CMP and GMP from the same mice. Similarly, using the MRP8-Cre-ires/GFP transgenic mouse and floxed JunB mice, the JunB allele was deleted in a subset of GMP and most, if not all, granulocytes, correlating with reduced levels of JunB mRNA in GMP and no detectable expression in granulocytes. An expansion of the granulocytic lineage was observed but no MPD. On the contrary, targeted inactivation of LT-HSC using Mix-Cre transgenic mice resulted in complete loss of the JunB allele and complete loss of JunB mRNA expression. This led to increased numbers of LTHSC and GMP but also to development of MPD [49]. A later paper by the same group [125] demonstrated that JunB disturbed HSC self-renewal while skewing differentiation to the myeloid lineage without causing LT-HSC exhaustion in the short term but with loss of quiescence. Skewing to the myeloid lineage appears to occur with increased speed as if abbreviating the intermediate stages of development. Transplantation of small numbers of JunB-deficient HSPC to irradiated recipients resulted in reduced reconstitution relative to controls. This was shown to be due to a fewer content of HSCs in bone marrow of JunB deficient animals correlating with decreased number of quiescent cells and increased fraction of S/G2/M in CD34- Flk2- LSK subset. When the G0 subset from this population was sorted and transplanted to irradiated recipients, hematopoietic reconstitution was again impaired. The CD34- Flk2- subpopulation itself displayed abnormal cell cycle distribution with predominance of G1/S/M phases. Staining with SLAM (signaling lymphocyte activation molecule) markers identified a CD150+ CD48- Flk2- quiescent LSK population identical in size in JunB deficient and controls and a CD48+ Flk2- non-quiescent LSK population which was 20 times expanded in JunB deficient animals. The CD48+ fraction, either from JunB deficient or control mice could not give rise to permanent reconstitution but that of JunB deficient gave transient myeloid expansion. However, junB deficient CD150+ CD48- Flk2- LSK cells displayed more repopulating capacity than control cells reflecting increased production of myeloid cells. These findings indicate that the reduction in repopulating potential observed when transplanting small numbers of LT-HSC is due to dilution of this population through expansion and differentiation towards a nonengrafting CD48+ population which is skewed to the myeloid lineage. Although JunB deficient LSK cells seemed to display normal regenerative potential also showed decreased quiescence and molecular analysis revealed downregulation of early G1 cyclins and increased expression of late G1 ((E1 and E2) as well as S-G2/M cyclins in JunB-deficient mice. In addition, loss of stemnes of this population should be a consequence of the observed decreased levels of p18 and p57. A trend towards decreased expression of Pten, Hoxb4 and Hoxa9, Notch1, Hes1 and Hes5 and normal levels of c-Myc and n-Myc was detected in JunB deficient cells. Nevertheless, quiescent JunB deficient LSK cells consistently displayed downregulation of Hes1 (a target of the Notch pathway which induces lymphoid lineage commitment) and the TGF-β targets Smad7, p57 and Hes1. Furthermore, Hes1 expression could not be induced in JunB deficient cells upon incubation with OP9DL1 stromal cells that express the Notch ligand delta1. In summary, JunB deficiency diverts normal hematopoietic differentiation towards the myeloid fate in reverse pattern to the role played by the Notch pathway that directs differentiation to the T lymphoid lineage. In the absence of Notch downstream target Hes1, precursor cells take the available myeloid route in increased numbers. The oncogenic pathway involved in these experiments seems to be the same described by Geserick et al. discussed above: repression of TGFβ by TERT. If JunB represses hTERT expression and through it, upregulates TGFβ it could be surmised that JunB deficiency has the opposite effect. However, it is still possible in this experiment that TGFβ could have been repressed by some other signal because Takakura et al. have reported that AP-1suppresses hTERT but it did not suppress TERT in mouse fibroblasts [6]. Here, however, species differences were not involved as only mice cells were used. In addition, leukemogenesis in this model could be driven by JunB deficiency acting on the mTERT gene in a way that does not impinge on the catalytic activity of telomerase but still could affect the immortalizing activity of mTERT as these activities can be dissociated. In any case, this model shows that oncogenesis mediated by TERT-TGFβ (or TGFβ derepression by JunB deficiency independent of TERT) signaling involves accelerated exit from the stem cell compartment. In other words, transformation seems to occur in the post-stem cell population. The telomerase immortalization activity and TGFβ inhibition, in mice as in humans might be triggered by JunB deficiency. These functions, coupled with downregulation of the Notch pathway, which is likely responsible for diverting differentiation into the myeloid lineage seem to be responsible for myeloid leukemia in this model. In most situations examined in this paper overexpression of TERT and TGFβ downregulation was hypothesized to be triggered by moderate telomere erosion and TASP. Here, TERT upregulation and TGFβ inhibition must be present in the model of Santaguida et al. in the HSC compartment but also in GMPs as well as in intermediate stages. However, they surmised that the HSC was the direct target of transformation. It seems, nevertheless, that leukemic transformation was not generated from within the most primitive fraction of LT-HSCs, that is, within CD150+ CD48- Flk2- cells but from the CD48+ that differentiate to myeloid cells. Cell differentiation and increased exit from the most primitive HSC compartment seems essential to transformation. It seems that transformation originated in this exit population which can retain markers of LT-HSC or even could re-express these markers after being transformed rather than on the most primitive CD150+ CD48- Flk2-. On the other hand, JunB inactivation has been detected in patients with different subtypes of myeloid malignancies likely originating in committed cells [125].

#### Neoplastic transformation downstream of the HSC in other oncogenic pathways

*Some findings in the PI3K-AKT pathway of leukemogenesis bear an interesting parallelism to leukemogenesis induced by JunB deficiency. In these experimental models AKT is usually activated in HSC by Pten deletion or constitutive myr-Akt constructs transduced to BM cells using the stem cell virus MSCV. Pten downregulation results in activation of the PI3 kinase pathway and AKT phosphorylation and activation. Invariably, Pten deletion leads to HSC mobilization and HSC exhaustion followed b tumorigenesis* [126, 127]*. BM cells containing a constitutively active form of Akt (the MSCV-IRES-GFP-myr-Akt construct) injected to syngeneic irradiated recipients induced an MPD which progressed to either AML or ALL. Despite the MPD, the proportion of GFP + LSK cells (enriched in HSCs and progenitors) were decreased compared to controls, consistent with HSC mobilization and/or apoptosis of HSCs* [128]***.*** *In contrast to these findings, Sykes* et al [129] *described suppression of growth of AML-AF9 leukemia by enforced constitutive Akt activation using an MSCV-IRES-GFP.myr-Akt construct. Suppression of growth was associated to myeloid maturation and myeloid maturation related cell death. Since this occurred also in the presence of rapamycin, Sykes* et al *surmised that Akt utilizes pathways other than mTOR activation for myeloid maturation (pAkt activates mTORC1 by relieving suppression exerted on mTORC1 by TSC2). As this is excluded under rapamycin treatment (as well as in raptor null cells) other actions of pAkt should be responsible for myeloid maturation and growth inhibition. pAkt phosphorylates and inactivates different substrates such as TSC2, GSK-3β and FOXO. Interestingly, pAkt Ser473 is dispensable for TSC2 or GSK-3β inactivation but is required for FOXO inactivation. Growth inhibition and myeloid maturation can be achieved by either inactivation or compound deletion of FoxO1/3/4. Nuclear FOXOs are essential to maintain quiescence and functional integrity of HSCs and are considered tumor suppressors as their deletion results in lymphoma development. FOXO inactivation by pAKT was associated to translocation of nuclear FOXO to cytoplasm. Therefore tumorigenesis in this system is delivered through FOXO. Since Akt promotes tumorigenesis the finding of decreased leukemic growth caused by enforced Akt was paradoxical. A possible clue to explain this paradox was the observation in leukemia initiating cells (LICs), which had the immunophenotype of GMPs, of a pattern of attenuated Akt activity (reduced pAktSer473 and pAktThr308) which is identical to that of normal HSCs and that appears to be essential to their self-renewal. The mechanism of self-renewal must, necessarily, have been coopted by these cells after they have left the stem cell compartment as a result of FoxO inactivation. Since all these changes (myeloid maturation, Foxo nuclear exclusion and inactivation are a consequence of Akt activation, the reappearance of the stem cell pattern of Akt activity during tumorigenesis can only be explained by the occurrence of a process similar to reprogramming (partial reprogramming?) in a GMP,* i.e. *Foxo inactivation by activated AKT has pushed a normal stem cell or more likely a progenitor cell into enhanced mobilization-differentiation and this cell converts by partial reprogramming into a LIC (displaying low AKT activation). It had previously been reported that the activity of AKT is attenuated in HSCs as required by their quiescence. The level of AKT activity rises in normal GMPs. However, the leukemia initiating cells (LICs) which in this model share the immunophenotype of GMPs (Lineagelow, c-Kithigh, FcγRII/III+, CD34+) exhibited, contrary to non-neoplastic GMPs, markedly reduced pAktSer473 and pAktThr308 in response to stimulation with SCF. Enforced expression of myr-Akt, induced myeloid maturation, as shown by forward and side scatter, and expression of mature myeloid markers (CD11b) accompanied by maturation related cell death and retarded growth. This interpretation is not at odds with the beneficial effect of myr-Akt on established leukemia by promoting myeloid maturation and reduced tumor burden because this effect is accomplished through apoptosis as well as maturation and concomitant proliferation arrest of bulk tumor cells. LICs could re-emerge or be preserved in this process. Reversibility of FOXO inactivation may help to maintain a balance between cancer stem cells and cancer cells. Tothova* et al [130] *showed that the physiological transition from HSC to myeloid cells follows a developmental program independent of FOXOs. Therefore, the non-physiological type of differentiation induced by FOXOs inactivation may be relevant for leukemogenesis.*

*In the T lymphocyte lineage, it has been shown that FoxO inactivation precedes malignant transformation. Finlay* et al *demonstrated that Pten-null T cell progenitors cannot give rise to malignant lymphoma without inactivation of FoxO mediated by AKT phosphorylated at Ser473 by PDK1* [131]*. Transgenic mice harboring a Rictor deletion cannot phosphorylate AKT at Ser473 and are unable to inactivate FOXO which results in impaired leukemogenesis* [132].*. Rictor deletion also recued HSC depletion observed after Pten deletion. Magee* et al [132] *observed that AKT phosphorylation does not increase in HSCs that divide under physiological conditions.*

*The specific resistance of HSCs to leukemia development is also reflected on developmental-dependent differences in the response of hematopoietic cells to PI3K/AKT signaling. It has been shown that fetal and neonatal HSC are resistant to Pten-induced leukemia as well as to other pre-leukemic effects such as increased exit from stem cell pool and HSC exhaustion. Leukemia protection declines in parallel to ability of multilineage reconstitution from 4 week of age onwards. Interestingly, before this age, Pten-deleted HSCs did not exhibit increased AKT phosphorylation consistent with Pten independent regulation of AKT whereas cells in more mature developmental stages such as GMP-monocytes exhibit the adult pattern of response to Pten deletion* [132, 133]*.*

*The role of FOXO inactivation in leukemogenesis indicates that a differentiation step must precede development of the stem cell features that characterize the cancer stem cell while acquisition of stemness may be supported by TERT expression or another mechanism capable, like TERT, of inducing spontaneous conversion of committed cells to stem cells. The non-physiological type of differentiation induced by Foxos* [131] *may be required for this process. However, maintenance of stemness in the cancer stem cell may depend also on other mechanisms that are shared by normal stem and cancer stem cells. This is consistent with stemness of cancer stem cells being undermined by some of the same genetic alterations that compromise the stemness of normal stem cells. For instance, Bmi1 is essential for maintaining HSCs. In its absence, AML can be induced by retroviral introduction of Hoxa9 and Meis 1 oncogenes. However, Bmi1 deficient leukemic cells were unable to transplant the disease to secondary recipients* [74]*. A similar finding was observed in a model of PML/RAR acute promyelocytic leukemia in mice wild type or homozygous deficient for p21. Both congenic strains developed the disease but p21 deficient BM cells could not propagate the disease to secondary recipients. This defect correlated with a dramatic reduction in the number of cancer stem cells (p21 deficient LKS+ cells were reduced about 50-fold)* [74]*.*

*Finally, the finding by Sykes* et al *that FOXO depletion led to JNK/c-Jun activation may be of great significance. This inverse correlation suggests a compensatory response in order to block mobilization and differentiation of HSCs induced by FOXO deactivation implying a functional redundancy in both signaling pathways. As mentioned above the deletion of JunB (a member of the AP-1 family of transcription factors) in LT-HSCs results in loss of quiescence with expansion of a CD48+ Flk2- non-quiescent population and increased production of myeloid cells as well as development of MPD mimicking FOXO inactivation.*

*The discovery of the regulatory role of Id1 on AKT signaling* [134] *support the former contentions The level of pAKT increases in parallel with Id1. This is consistent with the increased myeloid lineage commitment induced by both of these molecules and, in turn, confirms the claim that they act downstream of the HSC compartment.*

#### Other reports suggesting resistance of HSCs to malignant transformation

The ETV-RUNX1(also known as TEL-AML1) fusion protein generated by t(12;21)(p13;q22) is associated to 25–30% of all pediatric ALLs. When the CD34+ CD38- BM population of ALL patients harboring this translocation (phenotype of normal HSCs) was divided into two subgroups: a CD34+ CD38- CD19+ (which was the major population and a minor CD34+ CD38- CD19- group it was shown that the CD19+ subset which is not present in healthy individuals was the target of the translocation and the subpopulation which could give rise to leukemic cells. The ETV-RUNX1 fusion protein was not present in the more primitive CD19- subset. In addition myeloid CD34+ CD33+ CD19- did not contain the fusion protein whereas all CD34+ CD33- CD19+ pro-B cells contained the fusion protein (CD19 is a lymphoid marker). This conclusively proves that the translocation arose in B cell committed progenitors. In addition, the normal HSC compartment CD34+ CD38- CD19- was unaffected as it is of normal size and does not contain the fusion protein and could generate multilineage reconstitution The t(9;22)(q34;q11) is commonly associated to CML but it can also lead to ALL. Both p210 and p190 BCR-ABL fusion proteins may occur in ALL depending on the specific breaking point on the BCR gene. Although in p210 t(9;22) ALL, most CD34+ CD38- CD19- as well as CD34+ CD38- CD19+ cells contained t(9;22) suggesting that the translocation is already present in the most primitive compartment, only the CD19+ subpopulation can give rise to leukemic engraftment whereas CD19- cells were capable of multilineage reconstitution. As for the p190 BCR-ABL findings were similar to those on ETV-RUNX1 and confirmed a progenitor B cell origin of the translocation. Although it might be claimed a stem cell origin for p210 BCR-ABL, based on the presence of the chromosomal translocation in this compartment, it was clearly shown that malignant transformation did not arise until cells had transited to a later differentiation stage. Moreover, while CD19+ but not CD19- progenitors from either ETV-RUNX1 or p190 BCR-ABL can give rise to leukemic reconstitution, the CD19- (uncommitted) cells of p210 BCR-ABL ALL, in spite of harboring the chromosomal translocation, gave rise, when transplanted to NOD-SCID mice, to normal multilineage human hematopoiesis. This striking observation is consistent with a special refractoriness of stem cells to malignant transformation and may even suggest a capacity for healing residing in the stem cell compartment. In addition, these researchers observed that HSC niches were not affected by the leukemic process which would be the case if leukemia would arise within this compartment [135].

Another suggestion on the specific resistance of HSCs to leukemic transformation comes from the result of a study on the effect of overexpression of BCL-2 in hematopoietic cells (including HSCs). Transgenic mice expressing BCL-2 under the H-2Kb promoter contained increased numbers of HSCs which were more quiescent and showed increased repopulation potential. Some myeloid and lymphoid leukemias developed in transgenics but no stem cell leukemias were described in spite of the high level of expression of BCL-2 in the HSC population. No stem cell leukemias were described in this report, in line with similar findings in which BCL-2 was under the control of other promoters [3].

#### Resistance to direct transformation of stem cells in non-hematopoietic tissues

An example of stem cell resistance to tumor development from a non-hematopoietic tissue is suggested by the topdown theory of intestinal carcinogenesis [136].

A report from Grigoriadis et al. [137] suggests resistance of bone stem cells to tumorigenesis through an unknown mechanism. Homozygous transgenic mice expressing c-fos fused to the murine H-2 kb class I MHC promoter were shown to develop osteosarcomas with 100% penetrance and short latency. Heterozygous mice develop osteosarcomas after 6 month of age. Exogenous fosB and c-Jun did not cause bone abnormalities. It was shown that c-fos is the component of AP-1 which specifically alters the phenotype of bone tissue. The expression of exogenous c-fos is not observed during embryonic development or in newborn mice. Its expression first occurred between the 2nd and third week of age shortly before tumor development. Then it is detected in osteoblasts lining bone surfaces and osteocytes embedded in the bone matrix. On the contrary, endogenous c-fos is expressed during embryonic life as well as newborns. By IH analysis and RNA hybridization, the endogenous gene expression was detected in osteoblasts lining bone surfaces and in chondrocytes of the articular surfaces and the epiphyseal growth plates. Interestingly, mRNA from the exogenous c-fos was detected in tumor cells and osteocytes but not in chondrocytes of articular surfaces or growth plate chondrocytes. JunB and JunD were also ubiquitous in tumours. The articular surface and epiphyseal growth plates contain the bone stem cells that generate new bone during growth, with a similar function to the basal cells of lining epithelia in tissue replacement. In other words the oncogenic form of c-fos is specifically not expressed in bone stem cells. This fact suggests a specific resistance of bone stem cells to the tumor-promoter exogenous c-fos.

##### Suggested quantitative relationship between telomerase expression and immortalization

A final aspect of the cell-autonomous mechanism linked to telomerase signaling is worth mentioning: the number of immortal cells in a given tumor has been suggested to be dependent on the level of telomerase activity detected. Telomerase activity was higher in small-cell lung carcinoma than in non-small cell lung cancers. It was detected in 100% of small-cell lung carcinomas with altered telomere length compared to 80% of primary lung cancers [138]. Interestingly massive apoptosis of tumor cells has been observed in lymph node metastasis of small-cell lung carcinoma in terminal phase [139]. This paradoxical association of massive apoptosis and tumor aggressiveness can only be explained if apoptosis is unable to prevent tumor growth due to the high proportion of cancer stem cells within the tumor population. Rb is frequently mutated in small cell lung carcinoma and this mutation might be linked to a highly immature stage of neoplastic transformation which may be associated to widespread expression of telomerase (to be discussed below).

In childhood B cell precursor ALL, leukemic cells were observed in immature as well as mature tumor populations. This finding was interpreted as supporting an stochastic model rather than the LSC model of tumor organization [2]**.** The present hypothesis allows for phenotypically mature populations with properties of cancer stem cells.

#### Parallelism between hematopoietic and epithelial cancers

It has been commented above that Id-1 antagonizes lymphoid-lineage promotion and myeloid-repression induced by E2A. Bone marrow cells transduced with an Id-1-expressing retrovirus remained in culture for over 1 year without undergoing terminal differentiation (a majority of cultured cells were myeloblasts with some promyelocytes and myelocytes). Upon transplantation these cells induced an MPD-like disease which could not be transplanted to secondary recipients.

Id-1 has also been associated with epithelial cancer. Promotion of breast cancer was attributed to the ability of Id-1 to expand the mammary stem cell pool while repressing differentiation. However, a closer look may suggest that cancer promotion by Id-1 may be linked to its overexpression at early post-stem cell stages in line with the aforementioned overexpression of Id-1 in common myeloid progenitors and its ability to induce myeloid lineage expansion. In breast, overexpression of Id-1 in MMTV-Id-1 transgenic mice led to increased incidence of breast tumors of basal subtype (2 out of 69 at 24 months of age). Id-1 was shown to cause expansion of mammary stem cells (CD49^high^) as well as its downstream progeny as indicated by increased ductal size and larger extension of epithelial outgrowths. Deficient terminal differentiation was also detected with high expression of basal markers. Breast cancer promotion by Id-1 was shown to be mediated by the Wnt/c-myc pathway as pharmacological inhibition of Wnt/c-myc blocked tumorsphere formation in Id-1 overexpressing MCF7 cells and acceleration of tumorigenesis was accompanied by increased c-myc expression [140]. However, contrary to the common assumption that cancer promotion involves the action of this pathway on stem cells, it can be argued that this pathway increases mobilization of the stem cell pool which leads to exhaustion of its regenerative potential with concomitant replicative stress on the post-stem and progenitor population. In addition restrained differentiation may allow for longer cell turnover with possible telomere damage and/or aberrant telomerase expression through the ability of c-myc to induce telomerase. Moreover, partial suppression of differentiation may mask the real extent of the stem cell pool. Multiple studies suggest that c-myc promotes exit from the stem cell compartment. Loss of c-myc function in mice resulted in expansion of hematopoietic stem cells (HSC) and diminished progenitors with pancytopenia and bone marrow devoid of c-myc activity reconstitutes HSCs but not more mature lineages. In contrast, the inability to downregulate c-myc resulted in increased differentiation at the expense of self-renewal [141]. Studies of c-myc expression in skin essentially reproduce those decribed in the hematopoietic system. A neoplastic although reversible phenotype upon overexpression of Myc in suprabasal layers was not described in experiments where Myc expression was targeted to the basal layer using the keratin 14 promoter [142].

Kim et al. [143] showed that c-Myc drives a program common to embryonic stem (ES) and cancer stem (CS) cells but distinct from the core stem cell targets. Nakagawa et al. [144] reported that the promotion of iPSC generation by Myc is independent of its transformation property and that both chimera and progenies derived from mouse iPSC have an increased incidence of tumor formation due primarily to reactivation of the c-myc retrovirus.

Simple expansion of the stem cell pool by different procedures including Wnt3 expression has not been related to greater cancer incidence [7].

Collectively, these data incriminate an early-stage post-stem cell population rather than the stem cell as the target of the Wnt/myc tumor promotion oncogenic pathway. Not surprisingly, the above considerations fit well with the above suggested division on two main cancer subtypes according to early vs late stage of cell of origin. It has been established that two distinct transgenic models of breast cancer, MMTV-NeuYD and MMTV-Wnt1 reflect different forms of human breast cancer pathophysiology characterized by early vs late stage morphology and differentiation markers. The Neu oncogene induces tumors that exhibit uniform luminal histology, require CDK4 and cyclin D1 for transformation and are thought to reflect the ErbB2 subtype of human cancer. In contrast, Wnt1-driven tumors exhibit features in common with human basal subtype that falls within the subgroup of TN (triple negative tumors) (lack ER and PR expression and Her2 gene amplification. Id-1 and Id-3 expression is seen in tumor cells of Wnt-1 driven tumors but not in Neu-induced tumors [145].

#### Do some early cancers arise directly from stem cells? Telomerase signaling in other early cancers

*Knowledge of the differentiation ladder in hematopoietic system has been made possible by available assays that can asses repopulating ability at every stage of development. These assays confirm unambiguously that only LT-HSCs are endowed with indefinite self renewal ability shared only with cancer stem cells. Unfortunately, there are no similar assays that could establish a similar precise correlation between tissue development and loss of indefinite regenerative potential in other tissues. This fact must be remembered when analyzing early childhood tumors that arise during retinal development (retinoblastoma), migration of cerebellar layers (medulloblastoma) or from undefined populations of neural crest origin (neuroblastoma). Assigning loss of self-renewal to commitment to a particular cell fate cannot be made with the same degree of confidence in neural as in hematopoietic tissue. Thus, although we have argued for a non-stem cell of origin of such tumors in a former paper* [22] *it cannot be discarded that early post-stem cells in neural tissue have preserved indefinite self-renewal potential. Certainly, in retinal development, commitment is already detected at the early progenitor cell stage as progenitor cells present during early retinal development give rise only to early-born cell types (ganglion cells and cones) whereas late progenitor cells give rise only to late-born cell types (bipolar cells and Müller glia). The different views concerning the stage of cell origin of retinoblastoma are reflected in two alternative models: the progenitor cell model and the transition cell model. The origin of retinoblastoma in a stem cell or a post-stem cell endowed with indefinite self-renewal is a likely possibility and most retinoblastomas contain a mixture of several cell progeny types which has been attributed to a multipotential cell of origin* [146]*. RB inactivation, the genetic lesion underlying retinoblastoma development, results in release and de-repression of E2F1 transcription factor, a key regulator of proliferation and apoptosis. E2F1 can target FOXO forming a complex that controls E2F1 proliferation and apoptotic function* [147]*. This fact links PI3-AKT and E2F1 signaling. E2F1 which has also been found to be strongly expressed in primary neuroblastomas and can activate Bmi1, which as stated above may in turn induce telomerase upregulation* [148]*. The sequence of E2F1-FOXO interaction has not been sufficiently studied and is not known whether it does induce exit from the stem cell compartment as it does in the leukemia model of Sykes* et al *(described above). Another interesting possibility is that this signaling could impinge on the very mechanism of telomere/telomerase regulation that we postulate can protect stem cells from transformation. As a matter of fact, it has been proposed that Rb family members could have a role in regulating telomeres independent of their role in cell cycle control* [149]*. A stem cell of tumor origin cannot be discarded in these tumor types.*

#### Complexity of stem cell protection to tumorigenesis

There are reports indicating the involvement of non-cell autonomous mechanisms that suppress tumor development through stem cell microenvironment signaling [150–152]. The fact that, in these reports, the suppression of breast cancer is mediated by either a regenerating or an embryonic mammary gland microenvironment suggests that tumor suppression could depend on the action of stem/primitive cells. Similarly**,** a protective anticancer role on bone marrow breast metastasis has been shown to be mediated by the myeloid/osteoclast progenitor (M/OCP). The anti-tumor effect of this cell appears to be linked to its undifferentiated stage as it is antagonized by the differentiating effect of granulocyte-colony stimulating factor which does not have any effect on osteoclast differentiation [153] It is conceivable that cell-autonomous and non-cell-autonomous mechanism may converge in order to protect stem cells from cancer development.

## Conclusion

*It is likely that most of the cancer models described might share a common late event in their oncogenic pathway which is a common disturbance of some function/s of the telomere complex.* The developmental stage of the target cell modulates the differential involvement of the telomere complex functions in transformation i.e., telomerase overexpression in early stage tumors versus telomere damage in tumors of late stage origin (apparently as an intitiating event). However, the telomere complex functions in oncogenesis may be related to its canonic functions but can also be independent of them since they can occur in the absence of enzymatic activity [111] and, conversely, it has been shown in humans that an hTERT mutant made by altering the carboxyl terminus of hTERT does not support telomere elongation or immortalization in spite of expressing TERT activity [154]. The present hypothesis lends itself to testing by genetic or pharmacological interventions that should impact differentially in tumors of early or late stage origin. On the other hand there are insufficiently known signaling pathways downstream of telomere/telomerase alteration, that can result in tumor promotion. As pointed out above Rap1 may connect signals arising from telomere alteration to several routes with oncogenic potential. It can activate NF-κB, a marker of leukemic stem cells absent in normal hematopoietic stem cells. NF-κB activation correlates with DOT1L activation which has been shown to be required for development of MLL-rearranged leukemias. Paradoxically, DOT1L induces transformation by upregulating HOXA9 and MEIS1 while in parallel it can impair somatic cell reprogramming (at least in ageing)**.** These apparently opposing effects of DOT1L appear contradictory [112, 113]. It is possible that re-expression of telomerase collaborates with DOT1L in order to overcome the barrier to transformation associated to impaired reprogramming and/or that an incomplete reprogramming induced by DOT1L is essential to the cancerous state. This double-edge effect of DOT1L, in promoting leukemia and antagonizing reprogramming harmonizes with the view of cancer transformation as an incomplete reprogramming event in a post-stem cell that maintains some phenotypic traits of its developmental stage of origin. We have seen above that Rap1 can also promote Foxo3a inactivation and that positive IKK staining, p-AKT and cytoplasmic Foxo3a correlated in 90 out of 130 human primary breast specimens. The IκB kinase complex (IKK) which is involved in NF-κB activation can also phosphorylate and induce nuclear translocation of FOXO3a independently of p-AKT and similarly to PTEN deficiency. *Other factors such as Id1 are also able to phosphorylate and activate AKT. On the other hand, promotion of tumorigenesis induced by inactivation of FOXOs in PI3-AKT signaling is associated to non-physiological differentiation which in the event of targeting a stem cell should cause it to leave* the stem cell compartment. A similar outcome could be the result of JNK/c-Jun inhibition.

*The scarcity of tumors directly derived from stem cells may be a consequence of the operation of some stem cell specific mechanisms, perhaps some type of telomere complex regulation specific to stem cells that could underlie the resistance of stem cells to transformation. In this context, genetic defects that impinge directly in the stem cell specific regulation of the telomere complex could explain possible exceptions to the inherent resistance of stem cells to neoplastic transformation postulated in this paper. Examples of this occurrence might be afforded by Beckwith-Wideman syndrome or retinoblastoma.* Nevertheless, even in these instances direct origin of cancer in stem cells has not been established. In addition there are reports indicating that stem cells are also protected from tumorigenesis by cell extrinsic mechanisms.

## Data Availability

Not applicable.

## Abbreviations

HSC: hematopoietic stem cell
LT-HSC: long term HSC
ST-HSC: short term HSC
LTR-HSC: long term repopulating HSC
MPP: multipotent progenitor
LMPP: lymphomyeloid progenitors
CLP: common lymphoid progenitor
CMP: common myeloid progenitor
GMP: Myelomonocytic progenitor
LSC: leukemia stem cell
LIC: Leukemia initiating cell
AML: acute myeloid leukemia
CML: chronic myeloid leukemia
PML: promyelocytic leukemia
APL: acute promyelocytic leukemia
MkEps: megakaryocytic-erythroid progenitors
ETPs: early thymic progenitors
ETP-ALL: early T cell leukemias
AMKL: acute megakaryoblastic leukemia
CMML: chronic myelomonocytic leukemia
MDS: myelodisplastic syndrome
L-GMP: leukemic granulocyte monocyte precursor
SCL/TAL1: stem cell leukemia gene
TCR: T cell receptor
BCR: B cell receptor
ATL: acute T leukemia
T-ALL: T-acute lymphoblastic leukemia
DP thymocytes: double positive thymocytes
DN: double negative thymocytes
GCSFR: granulocyte colony-stimulating factor receptor
GM-CSF: granulocyte-macrophage-colony-stimulating factor
BrdU: 5-bromodeoxyuridine
AP-1: a heterodimeric complex of the Jun (c-Jun, JunB, or JunD) and Fos (c-Fos, FosB, Fra-1 or Fra-2) family proteins
LIF: leukemia inhibitory factor
TERT: telomerase reverse transcriptase
TERC ,TR: the RNA component of telomerase
TA: telomerase activity
ALT: alternative lengthening of telomeres
cdk4R: (a point mutant of cdk4 unable to bind p16 which retains cyclin-dependent kinase activity
NF-κB: Nuclear factor-κB
OSKM: reprogramming factors: OCT4, SOX2, KLF4 and c-Myc
SASP: senescence-associated-secretory-phenotype
TASP: telomere-associated-secretory phenotype
TRF2: a member of the sheltering complex of telomeres
IKK: IκB kinase
NOD/SCID: nonobese diabetic-severe combined immunodeficiency
TGFβ: transforming growth factor β
Ca1h DNR: dominant negative TGFβ type II receptor
SP fraction: side population enriched in stem cells
TCR: T cell receptor
LSK: lineage -, c-Kit +, Sca-1 +, phenotype attributed to HSC and MPP
IgH: heavy chain Ig
Igκ: light chain kappa
STAT5: signal transducer and activator of transcription 5
STAT5-CA: STAT5 constitutively active
V(D) J: recombination
DSBs: double strand breaks
Flt3ITD: Flt3 internal tandem duplication
TCR: T cell receptor
BCR: B cell receptor
ATL: acute T leukemia
T-ALL: T-acute lymphoblastic leukemia
DP thymocytes: double positive thymocytes
DN: double negative thymocytes
Rap1: repressor/activator protein also known as Trf2IP; TERF2 interacting protein
HLH: helix-loop-helix
Id proteins: members of the helix-loop-helix family of transcription factors that competitively dimerize with other members of the HLH family (E2A, E2–2 and HEB) and inhibit their ability to bind DNA and activate specific genes required for differentiation. Ids can promote proliferation by inhibiting p16 and p21 as well as Rb
siRNA: short inhibitory RNA, shRNA, short hairpine RNA
M/OCP: myeloid/osteoclast progenitor

## Declarations

Ethics approval and consent to participate.
